# A Novel Mouse Synaptonemal Complex Protein Is Essential for Loading of Central Element Proteins, Recombination, and Fertility

**DOI:** 10.1371/journal.pgen.1002088

**Published:** 2011-05-26

**Authors:** Sabine Schramm, Johanna Fraune, Ronald Naumann, Abrahan Hernandez-Hernandez, Christer Höög, Howard J. Cooke, Manfred Alsheimer, Ricardo Benavente

**Affiliations:** 1 Department of Cell and Developmental Biology, Biocenter, University of Würzburg, Würzburg, Germany; 2 Max Planck Institute of Molecular Cell Biology and Genetics, Dresden, Germany; 3 Department of Cell and Molecular Biology, Karolinska Institute, Stockholm, Sweden; 4 Medical Research Council Human Genetics Unit and Institute of Genetics and Molecular Medicine, Western General Hospital, Edinburgh, United Kingdom; 5 Hefei National Laboratory for Physical Sciences at Microscale, School of Life Sciences, University of Science and Technology of China, Hefei, China; Stanford University, United States of America

## Abstract

The synaptonemal complex (SC) is a proteinaceous, meiosis-specific structure that is highly conserved in evolution. During meiosis, the SC mediates synapsis of homologous chromosomes. It is essential for proper recombination and segregation of homologous chromosomes, and therefore for genome haploidization. Mutations in human SC genes can cause infertility. In order to gain a better understanding of the process of SC assembly in a model system that would be relevant for humans, we are investigating meiosis in mice. Here, we report on a newly identified component of the murine SC, which we named SYCE3. SYCE3 is strongly conserved among mammals and localizes to the central element (CE) of the SC. By generating a *Syce3* knockout mouse, we found that SYCE3 is required for fertility in both sexes. Loss of SYCE3 blocks synapsis initiation and results in meiotic arrest. In the absence of SYCE3, initiation of meiotic recombination appears to be normal, but its progression is severely impaired resulting in complete absence of MLH1 foci, which are presumed markers of crossovers in wild-type meiocytes. In the process of SC assembly, SYCE3 is required downstream of transverse filament protein SYCP1, but upstream of the other previously described CE–specific proteins. We conclude that SYCE3 enables chromosome loading of the other CE–specific proteins, which in turn would promote synapsis between homologous chromosomes.

## Author Summary

Meiosis is a special type of cell division that takes place in the germ line of sexually reproducing diploid organisms. Major events during meiosis are the pairing, recombination, and segregation of homologous chromosomes. As a consequence, daughter cells are haploid and genetically diverse. Therefore, meiosis is of utmost importance for the life of sexually reproducing species as it maintains the species-specific chromosome number and generates genetic diversity within a species. Proper segregation of homologous chromosomes during meiosis requires homolog pairs to be physically linked. The synaptonemal complex (SC), a meiosis-specific structure conserved in evolution, is essential for this process. Defective assembly of the SC has deleterious effects on germ cells and can cause infertility in mice and humans. Here, we report on a newly identified protein component of the mammalian SC that we have named SYCE3. SYCE3 is strongly conserved among mammals. Using the mouse as a model system, we demonstrate that loss of SYCE3 leads to infertility in both sexes. Infertility is caused by disruption of meiosis due to the inability of *Syce3^−/−^* mice to assemble the central element of SCs. Our findings provide new insights into the complexity of SC assembly and its relevance to mammalian fertility.

## Introduction

Meiosis is a special type of cell division which gives rise to haploid, genetically diverse gametes. For organisms that reproduce sexually the correct haploidization of paternal and maternal genomes is of utmost importance. To ensure the correct separation of homologous chromosomes during anaphase I, homologs first have to find each other before coming into close physical proximity. This process takes place during prophase I of meiosis, which is highly regulated and can be subdivided into five stages: leptotene (chromosome condensation), zygotene (initiation of synapsis), pachytene (full synapsis), diplotene (desynapsis), and diakinesis (visible chiasmata) [1]. One key component that enables synapsis and crossover formation is the synaptonemal complex (SC), a largely proteinaceous, meiosis-specific nuclear structure. The SC consists of three components: two lateral elements (LEs), each of which are associated with a pair of sister chromatids, and a central region between the two LEs, that is composed of numerous transverse filaments and the central element (CE). The central region physically links homologous chromosomes in a zipper-like manner and thus mediates synapsis (reviewed in [2],[3]). The tripartite structure of the SC is strikingly conserved from budding yeast to humans emphasizing its prominent function during meiosis. Much of our current understanding in the field was obtained from organisms such as *Saccharomyces cerevisiae*, *Caenorhabditis elegans*, and *Drosophila melanogaster* [4]–[10]. However, the mouse also has advanced to a widespread model system for the analysis of SC composition and regulation [11], [12]. Investigations in the mouse are expected to provide a better insight into causes of infertility in humans [13]–[15]. Several mouse models have been generated over recent years in order to illuminate the process of mammalian SC assembly. With the aid of these a first molecular model of SC assembly, synapsis initiation and propagation was proposed [11].

SC assembly in mice is initiated during leptotene with the formation of the axial elements (AEs; i.e. the precursor structures of LEs) by the AE proteins SYCP2 and SYCP3 [2], [16]. The AEs colocalize with cohesin cores consisting of cohesin complex proteins [17]–[19]. Mice deficient for SYCP3 lack AEs and fail to form continuous cohesin cores [20]–[22]. Interestingly, knockout of the *Sycp3* gene results in a sexually dimorphic phenotype: SYCP3-deficient males are sterile due to massive apoptotic cell death during zygotene [20], whereas females are fertile, but exhibit a sharp reduction in litter size caused by aneuploidy resulting in embryo death in utero [21].

AEs become linked by numerous transverse filaments (hence, AEs are referred to as LEs) when synapsis is initiated in zygotene of wild-type mice. Transverse filaments (TFs) are mainly composed of SYCP1, a fibrillar protein with a central coiled-coil domain flanked by two globular N- and C-terminal domains [23]. SYCP1 molecules most likely form homodimers with a parallel orientation with both C-termini anchored in an LE and the N-termini interacting head-to-head in the CE with a SYCP1 dimer of the opposite LE [24]–. Two facts indicate the prominent function of SYCP1 during SC assembly: (1) disruption of the mouse *Sycp1* gene leads to sterility in both sexes, which is caused by massive apoptotic events during spermatogenesis and oogenesis. Detailed analysis of *Sycp1* null mice revealed a complete breakdown of synapsis of homologous chromosomes, although the mice show normal AE formation and chromosome alignment. Furthermore, SYCP1 is required for crossover formation and the repair of DNA double-strand breaks (DSBs) [27]. (2) When expressed in a heterologous system SYCP1 molecules have the capability to self-assemble and form structures that closely resemble SCs (i.e. polycomplexes; [26]). Therefore, SYCP1 may function as a molecular framework to which other proteins attach to accomplish SC assembly and progression of recombination events ([26], [27]; and below).

Because of the prominent function of SYCP1 in meiosis and its relevance for fertility, the identification and characterization of its interaction partners has received intense interest over the past years. Of particular interest are three proteins, SYCE1, SYCE2 and Tex12, which exclusively localize to the CE and, interestingly, contain a coiled-coil domain, which constitutes a common protein interaction motif [28], [29]. These proteins, together with SYCP1, form a complex in the CE. SYCE1 and SYCE2 were found to bind to each other and to SYCP1, whereas Tex12 forms a complex with SYCE2 and, therefore, indirectly interacts with SYCP1. The hypothesis that SYCP1 serves as a molecular framework is supported by the fact that disruption of the central region by eliminating *Sycp1* results in a mislocalization of all three CE-specific proteins [29]. In addition, knockout models of each of the three known CE proteins revealed that loss of either of these alters higher order polymerization properties and localization of SYCP1 [30]–[32]. This indicates that the in vivo relationship between SYCP1 and all currently known CE proteins is reciprocal.

Correct SC assembly is required for proper meiotic recombination and conversely, recombination is essential for correct SC assembly. This is particularly well documented in mice deficient for *Spo11*, which are characterized by the lack of Spo11-dependent DSBs. In these mice SC formation is much reduced or SCs form between non-homologous chromosomes [33], [34]. Thus SC assembly and meiotic recombination are mutually dependent on each other.

Here we report on the characterization of SYCE3, a novel protein specifically located in the CE of the mammalian SC. SYCE3 is exclusively expressed during male and female meiosis and colocalizes with SYCP1 and SYCE1. We show that SYCE3 is part of the previously described CE complex [28], [29] where it interacts with SYCE1 and SYCE2. To gain more insights into the function of SYCE3 we generated a *Syce3* knockout mouse. These mice are characterized by infertility in both sexes as well as complete disruption of synapsis and mislocalization of previously described CE proteins, indicating that SYCE3 is required for synapsis initiation and chromosomal loading of the other CE proteins (i.e. SYCE1, SYCE2 and Tex12). Furthermore, we demonstrate that loss of SYCE3 has no influence on the initiation of meiotic recombination, but is required for its progression.

## Results

### Identification and Characterization of SYCE3

**Figure 1:**
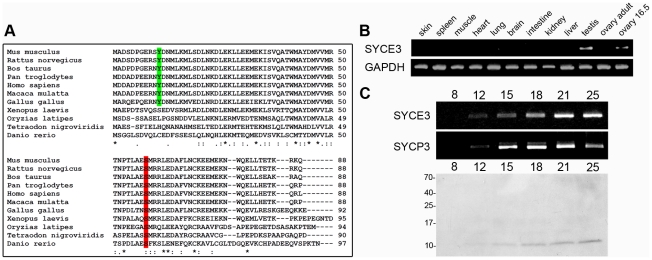
Identification and characterization of mouse SYCE3. (A) Multiple alignment of full-length SYCE3 protein sequences of various vertebrates. A predicted tyrosine phosphorylation site is marked in green, and a conserved serine phosphorylation site in red (calculated for the mouse protein sequence). (B) Tissue-specific expression pattern of SYCE3 mRNA as shown by RT-PCR analysis with SYCE3 specific oligonucleotides. (C) Temporal expression pattern of SYCE3 during mouse spermatogenesis analyzed by RT-PCR using RNA of testicular cells from mice of different ages (day 8–25) and Western blot analysis using 5×10^5^ cells of the same mice separated on a 16%/6 M urea tricine-SDS gel and detection of SYCE3 with affinity purified guinea pig anti-SYCE3 antibody.

We selected a set of genes identified by means of (1) predominant expression in testis and (2) a predicted nuclear localization, as well as a coiled-coil domain, within the encoded protein sequence from a gene expression profile initially used to elucidate the impact of *Dazl* knockout on gene expression in the developing gonads [35]. Using RT-PCR we demonstrated the selective expression of one of these genes -1700007E06Rik [35] - in adult testes and embryonic ovaries, and its absence in somatic tissues (Figure 1B). 1700007E06Rik encodes a protein (which we have named SYCE3) consisting of 88 amino acids. SYCE3 can be found in all vertebrate classes from fish to human (Figure 1A). It is highly conserved among mammals with an identity of 90% (96% similarity) at the amino acid level between mouse and human. PSORTII and SMART prediction of protein structural motifs revealed that SYCE3 contains a short coiled-coil motif (amino acids 6–39) which is conserved in all analyzed vertebrate sequences except for fish.

In order to determine the exact temporal expression pattern of SYCE3 mRNA during spermatogenesis, we performed additional RT-PCR experiments with whole mRNA fractions of pubertal mice testes of different ages. Analysis of the first wave of spermatogenesis revealed that SYCE3 mRNA is first detectable on day 12 (i.e. the onset of the prophase I of meiosis) and persists in older animals. Comparison of the temporal expression profile of SYCE3 mRNA with that of SYCP3 revealed a striking similarity, suggesting that SYCE3 also functions during the prophase I stage of meiosis (Figure 1C). To analyze SYCE3 expression at the protein level, we generated two antibodies against the full-length protein (see materials and methods). Western blot analysis of total protein fractions of pubertal mouse testes of different ages revealed a single band with the expected mass (12 kDa) from day 12 onwards, validating the fact that SYCE3 is first expressed with the onset of meiosis (Figure 1C). In addition, SYCE3 restriction to meiotic cells was confirmed by immunofluorescence analysis on frozen mouse testis sections: SYCE3 expression is confined to spermatocytes and completely absent in spermatogonia, spermatids and spermatozoa (Figure S1B).

### SYCE3 Selectively Localizes to the Central Element of the Synaptonemal Complex

**Figure 2:**
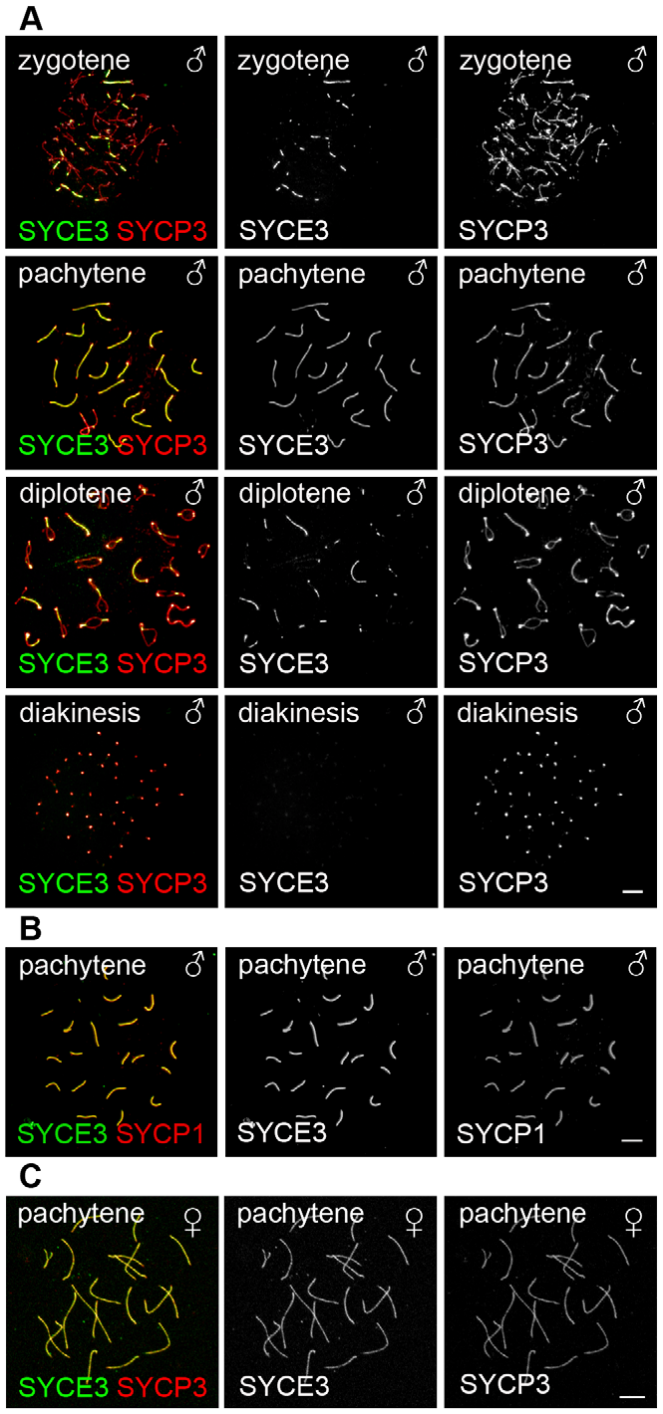
SYCE3 selectively localizes to the synapsed areas of homologous chromosomes. (A) Mouse spermatocytes in zygotene, pachytene, diplotene and diakinesis stages stained for SYCE3 (green) and SYCP3 (red). Bar, 10 µm. (B) Pachytene spermatocytes marked with SYCE3 (green) and SYCP1 (red). (C) Mouse pachytene oocyte labeled with SYCE3 (green) and SYCP3 (red). Images were acquired using a fluorescence microscope. Bar, 7.5 µm.

**Figure 3:**
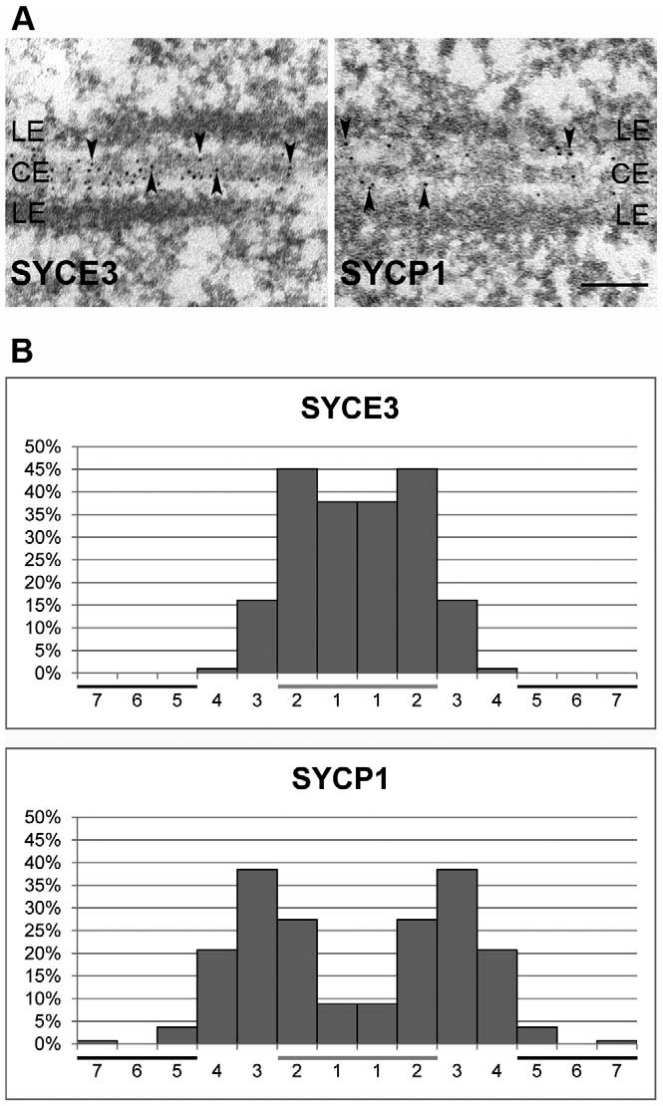
SYCE3 localization is restricted to the CE of the SC. (A) Immunoelectron microscopy of frozen rat testis sections marked with affinity purified rabbit anti-SYCE3 antibody (left panel) and a specific antibody directed against the coiled-coil region of SYCP1 (right panel). Arrowheads indicate gold-particles. Bar, 0.1 µm. (B) Quantification of gold-particle distribution in EM images of SYCE3 labeled (left, n = 304) and SYCP1 labeled samples (right, n = 135). The distance between the center of the SC and the LE was divided into seven sections and gold-particles were counted in each section. The CE of the SC is marked with a horizontal grey bar whereas the horizontal black bars correspond to LEs.

Using immunocytochemistry on spread mouse spermatocytes, we investigated the subcellular localization of SYCE3 in meiotic cells. For proper staging of meiotic cells we used SYCP3 as a marker for AEs/LEs (Figure 2A). Fluorescence staining of SYCE3 showed that it specifically localizes to the synapsed regions of homologous chromosomes, whereas SYCP3 was present in AEs and LEs. SYCE3 was first detectable during zygotene when synapsis is initiated and staining persisted on synapsed regions of homologous chromosomes until diplotene. The localization to synapsed regions was verified by double-labeling experiments performed with SYCE3 and TF-protein SYCP1 (a marker for synapsed regions) in which a virtually identical staining pattern was observed (Figure 2B). In pachytene oocytes SYCE3 also localized to synapsed chromosomes (Figure 2C). For a detailed analysis of SYCE3 localization within the SC we performed immuno-gold electron microscopy on testis sections using an affinity-purified SYCE3 antibody and an antibody against the coiled-coil region of SYCP1 as a control (Figure 3A). In preparations incubated with SYCE3 antibodies, gold particles exclusively localized to the CE of the SC. This is in strong contrast to the findings when using an antibody against the coiled-coil region of SYCP1, where - as expected - TFs between CE and LEs are labeled (see also [29]). For a quantitative analysis of immunogold data we sub-divided the distance between the SC center and the outer edge of LEs into 7 equal sections and counted the immuno-gold particles in each section (see [36]). In the case of SYCE3 the bulk of gold particles (n = 304) localized to the two sections adjacent to the SC center. In contrast, gold particles corresponding to the SYCP1 coiled-coil domain (n = 135) largely localized to the area between LEs and CE (see also [25]; Figure 3B).

**Figure 4:**
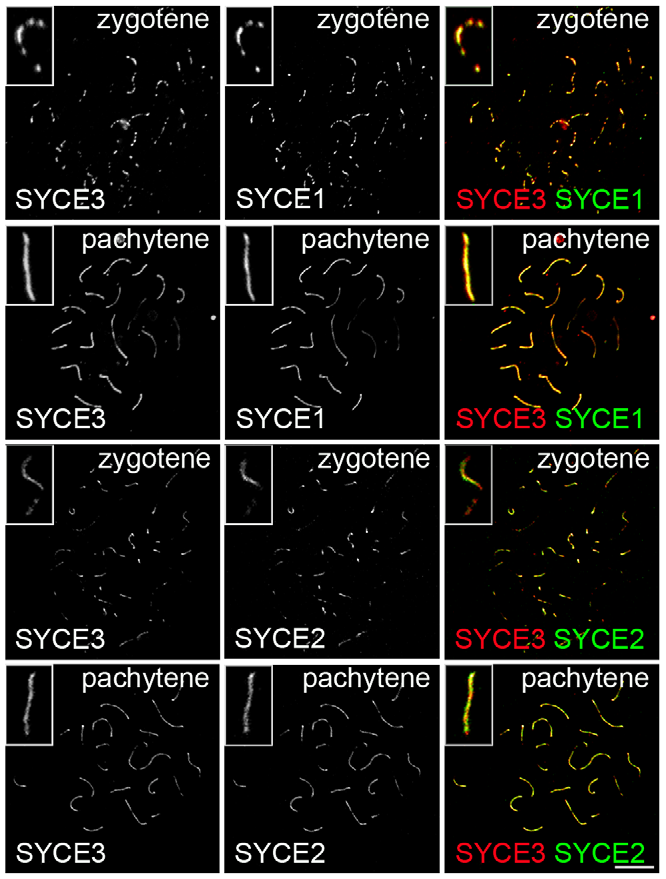
SYCE3 mirrors SYCE1 localization. Co-labeling of zygotene and pachytene spermatocyte spread preparations with SYCE3 (red) and SYCE1 or SYCE2 (green) acquired using a confocal microscope. Co-staining for SYCE3 and SYCE1 in zygotene and pachytene spermatocytes shows that these two proteins co-localize along the synapsed chromosomes (see inset). In contrast, SYCE3 and SYCE2 do not necessarily co-localize in zygotene and pachytene cells (see inset). Here, SYCE3 is distributed in a more continuous pattern whereas SYCE2 appears more punctuated. Bar, 10 µm.

Since three CE-specific proteins had been discovered previously (SYCE1, SYCE2 and Tex12; [28], [29]), we became interested in the localization of SYCE3 in respect to the other CE-specific proteins. As previously described, SYCE1 is distributed rather continuously in pachytene along synapsed areas of homologous chromosomes, whereas SYCE2 and Tex12 localize in a more punctuated pattern. Co-localization of SYCE3 and SYCE1 on spread mouse spermatocytes revealed that in both zygotene and pachytene cells these two proteins co-localize in a rather continuous pattern along the synapsed chromosomes (Figure 4, see inset). In contrast, double-labeling experiments with SYCE3 and SYCE2 showed that in zygotene and pachytene cells SYCE3 does not necessarily co-localize with SYCE2 (Figure 4, see inset). Here, SYCE3 was distributed in a more continuous pattern whereas SYCE2 appeared more punctuated.

### Lateral Element Assembly As Well As Central Element Components SYCE1, SYCE2, and Tex12 Are Not Required for SYCE3 Chromosome Loading

**Figure 5:**
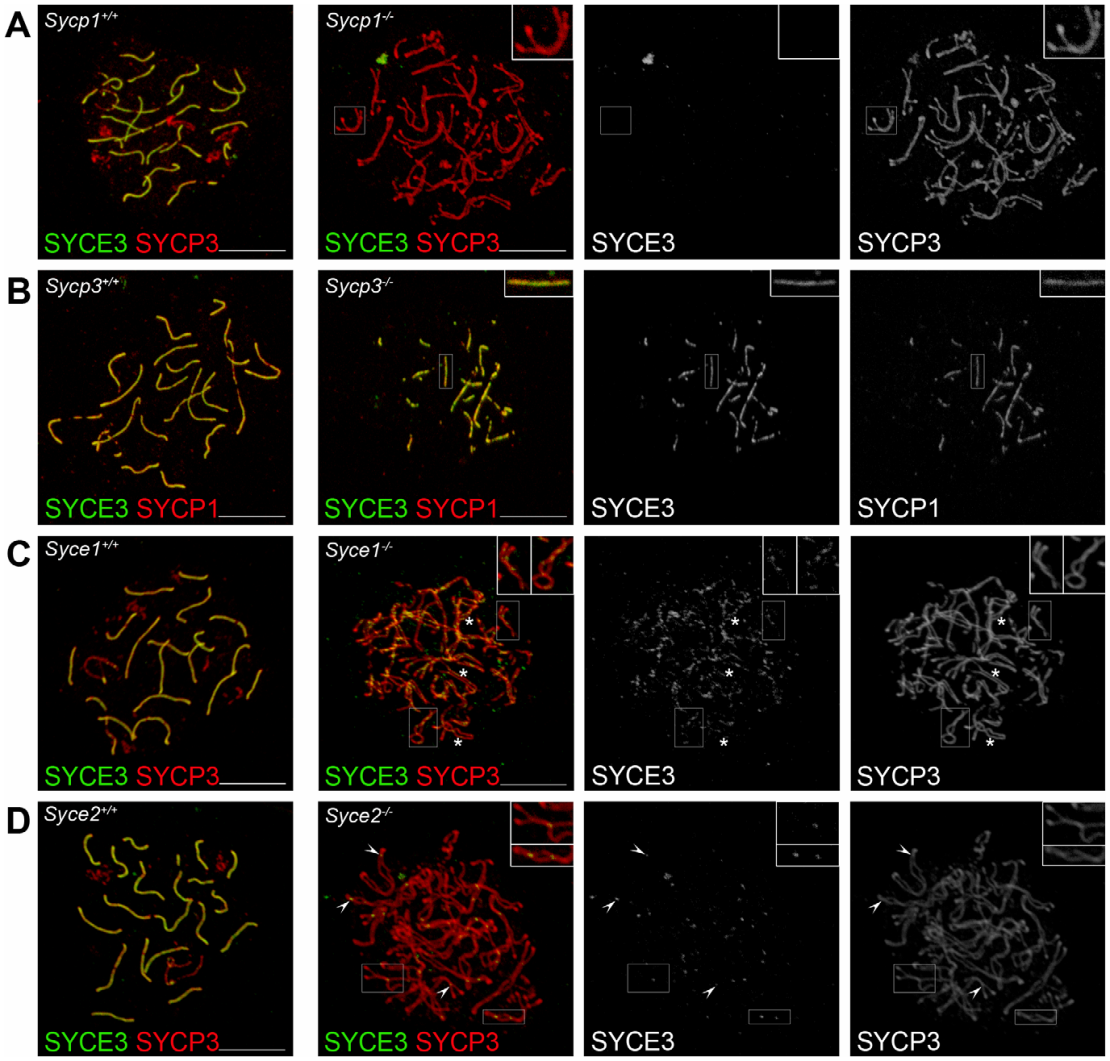
SYCE3 chromosome loading requires SYCP1 but is independent of SYCE1, SYCE2, and SYCP3. Confocal images showing the immunolocalization of SYCE3 (green) on spread preparations of (A) *Sycp1^−/−^* pachytene-like spermatocytes co-labeled with SYCP3 (red), of (B) *Sycp3^−/−^* spermatocytes co-stained for SYCP1 (red) and of (C) *Syce1^−/−^* and (D) *Syce2^−/−^* pachytene-like spermatocytes co-labeled with SYCP3 (red). In cells lacking SYCP1, SYCE3 loading is defective. In the absence of SYCP3 both SYCE3 and SYCP1 localize to synapsed regions. In cells deficient for SYCE1, SYCE3 localizes in a weak discontinuous pattern to AE independent of whether they are in close apposition or not (see inset). Here, asterisks mark SYCE3 staining on AEs that are not closely aligned. In *Syce2^−/−^* cells SYCE3 localizes to small foci corresponding to sites at which AEs are closely associated (see inset). Arrowheads point to sites of SYCE3 localization in *Syce2^−/−^* cells. Bars, 5 µm.

To investigate the possible dependence of SYCE3 localization on the presence of other SC-proteins, we performed immunocytochemistry on spread spermatocytes of mice deficient for SYCP1 [27], SYCP3 [20], SYCE1 [30], SYCE2 [31] and Tex12 [32]. In the absence of SYCP3, double-labeling for SYCE3 and SYCP1 showed that SYCE3 mimics SYCP1 localization, indicating that SYCE3 is loaded only to synaptic chromosome regions (Figure 5B). In *Sycp1^−/−^* mice, SYCE3 is completely absent from the AEs (Figure 5A). In the absence of CE protein SYCE1, on the other hand, SYCE3 localizes to the AEs in a weak discontinuous pattern which is independent of whether AEs are in close apposition or not (Figure 5C, and insets therein). In mice lacking CE protein SYCE2, SYCE3 localized to small foci at closely aligned chromosome axes (Figure 5D, and insets therein). As expected, SYCE3 also localized to small foci in mice lacking Tex12 (data not shown).

Taken together, the results described in the first part of this study lead to the conclusion that SYCE3 is a novel, meiosis-specific component of the CE of mammalian SCs. Chromosome loading of SYCE3 appears to require SYCP1, but no other currently known, CE-specific proteins.

### SYCE3 Is Required for Male and Female Fertility

**Figure 6:**
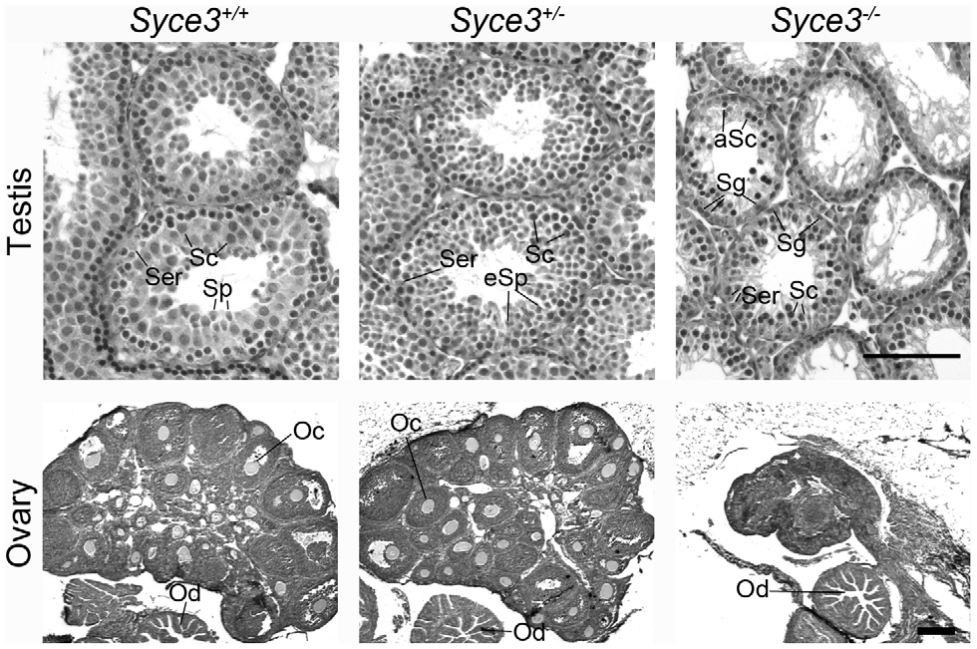
*Syce3* mutation results in disruption of meiosis. Histological analysis of testes (day 30) and ovaries (day 32) from *Syce3^+/+^*, *Syce3^+/−^* and *Syce3^−/−^* mice stained with haematoxylin and eosin. *Syce3^−/−^* testis lack postmeiotic stages whereas all stages of the spermatogenic cycle occur in the wild-type testis. The size of mutant ovaries is greatly reduced compared to wild-type littermates and additionally *Syce3^−/−^* ovaries are completely depleted of follicles. (Ser) Sertoli cells, (Sc) spermatocytes, (Sp) spermatids, (eSp) elongated spermatids, (aSc) apoptotic spermatocytes, (Sg) spermatogonia, (Oc) oocyte, (Od) oviduct. Long and short bars, 100 µm and 175 µm, respectively.

To gain deeper insights into the function of SYCE3, we generated a mouse strain lacking the SYCE3 protein. To this end, we replaced the two exons coding for the full-length protein with a neomycin cassette by electroporating a modified pKS*loxP*NT vector for gene-replacement into R1/E ES cells (Figure S2A; [37]). Using PCR and Southern blot we identified one positive ES cell clone (Figure S2B, S2C), which was injected into blastocysts of a C57BL/6 mouse to generate chimeric animals. Mating of chimeras resulted in heterozygote animals, which produced offspring with the mutated locus in Mendelian ratio. Correct deletion of *Syce3* in heterozygote and homozygote animals was confirmed by Southern blot analysis (Figure S2D). *Syce3^−/−^* mice displayed no overt somatic phenotype, but repeated mating attempts of wild-type with both *Syce3^−/−^* male and female mice did not produce offspring implying that both male and female *Syce3^−/−^* mice were infertile. Consistent with this, *Syce3^−/−^* testes of adult mice have a clearly reduced size compared to their wild-type littermates as previously reported for other infertile mice (Figure S2E; i.e. [27]). In addition, many TUNEL-positive meiotic cells (Figure S3A) and no postmeiotic cells were found in histological sections of *Syce3^−/−^* testes, indicating a defect during meiosis resulting in programmed cell death at stage IV (Figure 6 and Figure S3B). Compared to wild type females, ovaries of *Syce3^−/−^* littermates showed a sharp size reduction and lacked mature follicles suggesting a disruption of oogenesis (Figure 6).

### Initiation of Synapsis Is Dependent on SYCE3

**Figure 7:**
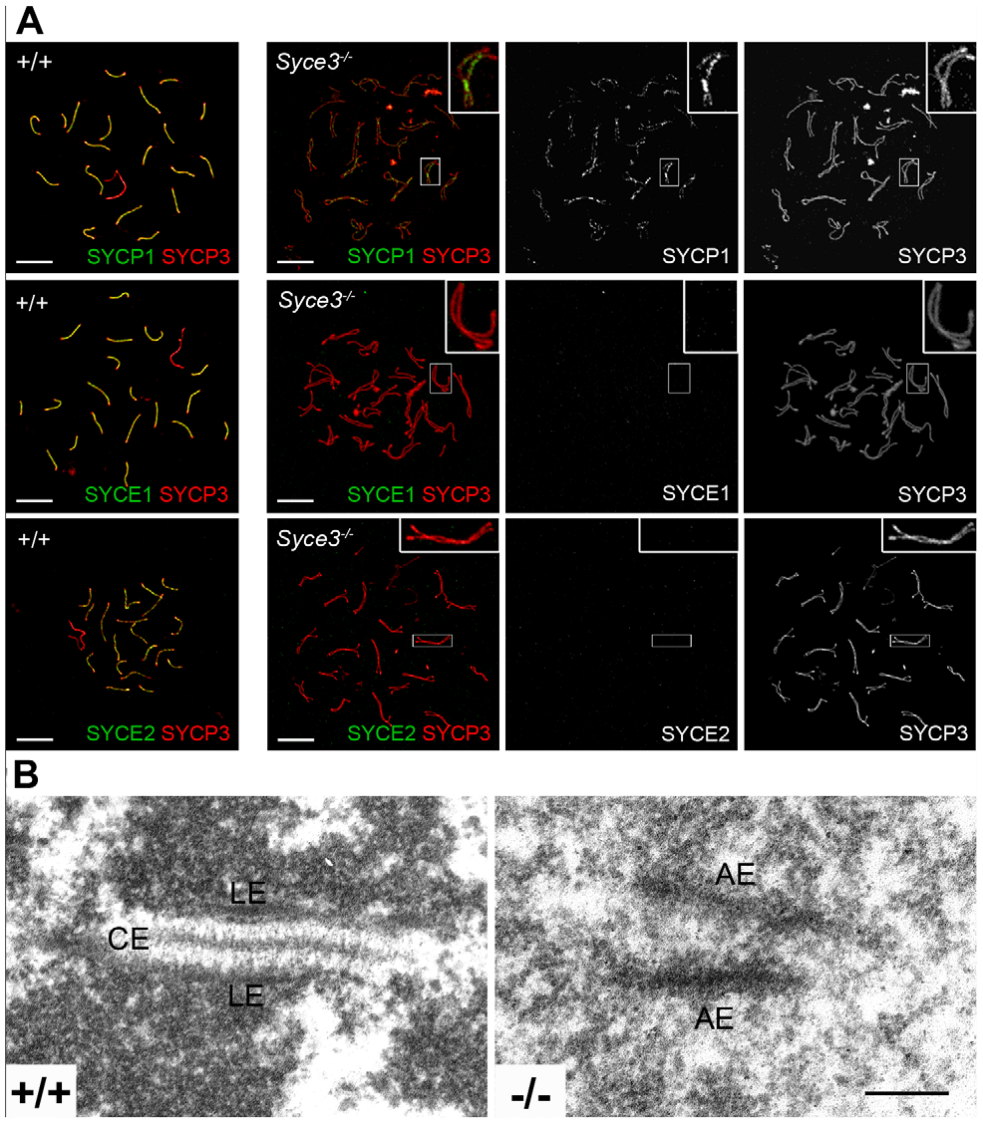
Initiation of synapsis is dependent on SYCE3. (A) Immunostaining of spread preparations of wild-type pachytene and *Syce3^−/−^* pachytene-like spermatocytes with SYCP3 (red) and SYCP1, SYCE1 or SYCE2 (green) acquired using a confocal microscope. In *Syce3^−/−^* spermatocytes, SYCP1 localizes in a weak discontinuous pattern at AEs independent of whether they are closely aligned or not. In contrast, SYCE1 and SYCE2 are completely absent from the AEs in SYCE3 deficient mice. Bars, 10 µm. (B) Electron micrographs showing synaptonemal complex assembly in wild-type and *Syce3^−/−^* spermatocytes. (LE) lateral elements, (CE) central element, (AE) axial elements. Bar, 200 nm.

To address the question why *Syce3* knockout leads to disruption of meiosis, we performed immunofluorescence analysis on spread mouse spermatocytes in which AEs were labeled with SYCP3 and TFs with SYCP1 as a marker for synapsis. As expected, in wild-type spermatocytes homologs were aligned in close juxtaposition during zygotene (data not shown) and full synapsis was achieved during pachytene (Figure 7A). In *Syce3^−/−^* pachytene-like spermatocytes the vast majority of AEs of the homologs were paired and aligned along their entire lengths. Incorrect alignment of autosomes was very rare (Figure 7A) and was most probably due to harsh chromosome spreading. Sex chromosomes on the other hand were frequently unpaired and appeared as univalents mimicking the phenotypes previously described for other CE proteins [27], [30]–[32]. In clear contrast to the wild-type situation, AEs completely failed to synapse in pachytene-like *Syce3^−/−^* spermatocytes (Figure 7A).

To further investigate the effects of SYCE3 depletion on synapsis, we compared SYCP1 localization in *Syce3^−/−^*, *Syce1^−/−^* and *Syce2^−/−^* spermatocytes. As previously described, in *Syce2^−/−^* cells SYCP1-staining is confined to regions where homologous chromosomes are in closer association (see Figure S4B and [31]). In contrast, in *Syce1^−/−^* cells SYCP1 localizes to the chromosome axes in a weak discontinuous pattern regardless of whether they are closely aligned or not (see Figure S4A and [30]). A similar distribution of SYCP1 was observed in cells deficient for SYCE3: here, too, SYCP1 was distributed in a weak, discontinuous pattern along the AEs, irrespective of whether they were closely aligned or not (Figure 7A). This is in strong contrast to wild-type spermatocytes, where SYCP1 localizes only to the synapsed areas of homologous chromosomes, but not to aligned AEs (Figure 7A). This suggests that SYCP1 is able to bind to the AEs via its C-terminus, and that the N-terminal interactions are impaired in the absence of SYCE3. Furthermore, these data clearly show that immunofluorescence analysis obtained under our experimental conditions can reproduce previously obtained weak immunofluorescence signals (compare Figure S4 and [30], [31]) and thus allow precise comparison of the different CE-mutant phenotypes.

We also investigated the localization of other CE proteins in *Syce3^−/−^* mice. To this end, we labeled spread *Syce3^−/−^* spermatocytes with SYCP3 as an AE marker in combination with either SYCE1 or SYCE2. Interestingly, both SYCE1 and SYCE2 were completely absent from the axes in cells lacking SYCE3 (Figure 7A). These results provide clear evidence that SYCE3 is required for loading of the other CE proteins.

To obtain more detailed information about synapsis defects in the *Syce3* knockout mice, we performed electron microscopic analysis on *Syce3^−/−^* testis. In wild-type spermatocytes, normal SCs composed of LEs with attached chromatin and a CE were observed. In contrast, we found partially aligned AEs in *Syce3^−/−^* spermatocytes, but no CE or CE-like structures at all (Figure 7B). This phenotype resembles the situation found in *Sycp1^−/−^* and *Syce1^−/−^* mice, but differs from that of *Syce2^−/−^* and *Tex12^−/−^* mice in which synapsis appears to initiate due to the assembly of short CE-like structures [30]–[32].

### SYCE3 Is Essential for Normal Progression of Meiotic Recombination

**Figure 8:**
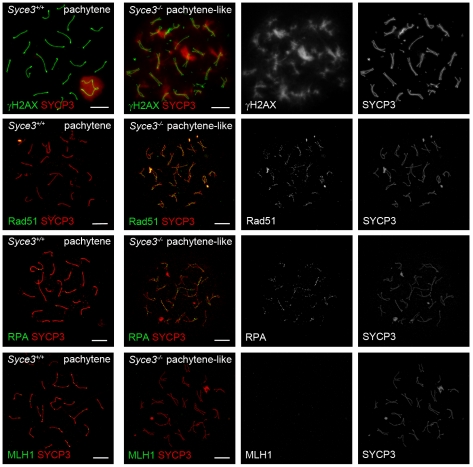
Complete repair of DSBs and crossover formation is not observed in *Syce3^−/−^* spermatocytes. Immunostaining of spread preparations of wild-type pachytene and *Syce3^−/−^* pachytene-like spermatocytes for γH2AX (red) and SYCP3 (green), RAD51 (green) together with SYCP3 (red), RPA (green) co-labeled with SYCP3 (red) and MLH1 (green) co-stained with SYCP3 (red). Images shown in row two to four were acquired using a confocal laser scanning microscope. Bars, 10 µm.

During leptotene, meiotic recombination is initiated by the introduction of DNA DSBs. These sites become marked by histone γH2AX. During leptotene and zygotene, γH2AX is located in large domains around the DNA breaks, but as meiotic prophase I progresses, it becomes restricted to the sex chromosomes [38]. In *Syce3* ^−/−^ spermatocytes, however, immunostaining of γH2AX revealed altered dynamics. While distribution of γH2AX in early (leptotene, zygotene) mutant spermatocytes resembles that of early wild-type cells (data not shown), γH2AX is not restricted to the sex chromosomes during the pachytene-like stage (Figure 8). Instead, γH2AX remains associated with most of the chromosomes in a cloud-like manner. The persistence of γH2AX-staining up to the advanced stages of prophase I suggests that DSBs are formed in *Syce3*-deficient spermatocytes, but that they are not efficiently repaired.

To gain additional insights into further processing of DSBs in mutant cells, we performed immunostaining for proteins that are specific for different recombination nodules. In wild-type mice, one distinguishes between early nodules (ENs) which appear prior to synapsis and assemble at sites of DSBs [39], [40], transitional nodules (TNs) during zygotene [41] and late recombination nodules (RNs) which mark sites of future crossover events [42]. ENs assemble at the leptotene stage and are made up of the two RecA homologs RAD51 and DMC1. They form numerous foci along chromosome cores and catalyze strand exchanges between homologous DNA molecules as a first step during processing of DSBs to crossover events [41],[43]. During zygotene of wild-type mice, RAD51 and DMC1 are gradually replaced by RPA. Thereby ENs are transformed into TNs which appear isochronously to synapsis. It is likely that TNs are involved in stabilization or resolution of early recombination intermediates [41], [43]. In *Syce3* ^−/−^ spermatocytes, RAD51 and RPA form numerous foci, localized to chromosome cores during zygotene (data not shown). This pattern of distribution resembles that of wild-type spermatocytes in early stages of prophase I (data not shown). These observations are consistent with the notion that early DNA-DNA interactions can be mediated by RAD51 and RPA in the absence of SYCE3. However, the following processing steps are likely to be disturbed as RAD51 and RPA remain associated with chromosomes in pachytene-like spermatocytes (Figure 8). MLH1 marks presumed future crossover sites [43]–[45]. Correspondingly, in wild-type pachytene spermatocytes each bivalent displayed one or two MLH1 foci. In contrast, male littermates lacking SYCE3 have no MLH1 foci, pointing to a disruption of crossover formation (Figure 8).

**Figure 9:**
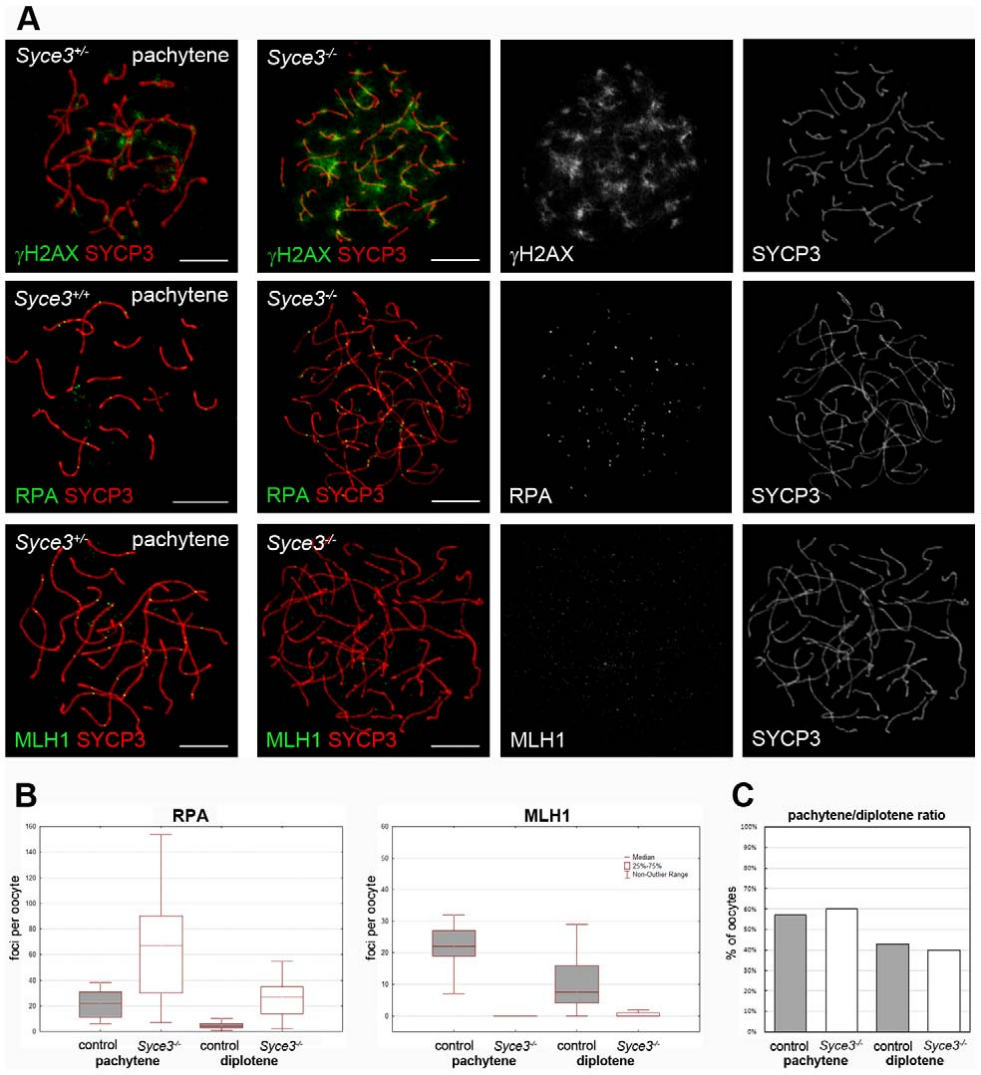
Complete repair of DSBs and crossover formation is not observed in *Syce3^−/−^* oocytes. (A) Immunofluorescence analysis on mouse oocytes collected from 19.5 dpc littermates. Wild-type and *Syce3^−/−^* oocytes double stained for γH2AX (first row), RPA (second row) or MLH1 (third row) and SYCP3 are shown. Images were acquired using a confocal laser scanning microscope. Bars, 10 µm. (B) Numbers of RPA foci (left) in pachytene (WT, n = 21; *Syce3^−/−^*, n = 27) and diplotene (WT, n = 15; *Syce3^−/−^*, n = 15) oocytes depicted in a box-and-whisker plot. Box-and-whisker plot of MLH1 foci (right) counted in pachytene (WT, n = 23; *Syce3^−/−^*, n = 26) and diplotene (WT, n = 18; *Syce3^−/−^*, n = 20) oocytes. (C) Pachytene/diplotene ratio of oocytes collected from control (n = 77) or *Syce3^−/−^* (n = 88) 19.5 dpc littermates.

To rule out the possibility that the persistence of RPA on chromosome axes and the lack of MLH1 foci in *Syce3*-deficient spermatocytes is due to their arrest in pachytene and their subsequent elimination by apoptosis rather than reflecting a direct function of SYCE3 in homologous recombination we additionally performed a close examination of recombination in *Syce3^−/−^* oocytes at 19.5 dpc (days post coitum). In concordance with earlier reports [46], [47] the majority of 19.5 dpc oocytes were staged at late pachytene or diplotene. Consistent with this, SCYP3 labeled AEs were fully synapsed in wild-type pachytene oocytes. As already described above for *Syce3^−/−^* spermatocytes synapsis was completely abolished in SYCE3-deficient pachytene-like oocytes. Compared to the situation in the male (see Figure 7A and Figure 8), however, pairing and alignment in 19.5 dpc *Syce3^−/−^* oocytes seemed to be more strongly affected (Figure 9A). Whether this finding reflects a female–specific function of SYCE3 in homologous pairing and/or alignment or is rather a secondary effect caused by defective synapsis cannot be judged at present.

Careful analysis of recombination markers in 19.5 dpc oocytes strongly supported the notion that SYCE3 is essential for progression of meiotic recombination (Figure 9A). Consistent with our findings in pachytene-like *Syce3^−/−^* spermatocytes, in 19.5 dpc oocytes of SYCE3-deficient animals γH2AX stayed associated with chromosome axes, unlike in oocytes of wild-type littermates (Figure 9A). This suggests the persistence of unrepaired DSBs. Moreover, as judged by quantitative analysis of RPA and MLH1 dynamics, processing of TNs to RNs appeared to be considerably affected by the absence of SYCE3: In wild-type oocytes the median of RPA foci per cell was 22 during pachytene and decreased to 4 at diplotene stage. At the same time, the median number of MLH1 foci per cell was 22 at pachytene and 7.5 at diplotene stage in the controls. Thus, both the dynamics of RPA and MLH1 foci as well as their relative abundance found in our wild-type controls were consistent with data reported previously [41]. In clear contrast, the median number of RPA foci in SYCE3-deficient pachytene-like oocytes was significantly higher compared to wild-type controls (67 vs. 22; p<0.001). Additionally, RPA foci persisted on chromosome axes of *Syce3* ^−/−^ oocytes at diplotene-like stage (median, 27 vs. 4; p<0.001) indicating that processing of TNs to RNs was impaired in the absence of SYCE3 (Figure 9B). In line with this notion, MLH1 was virtually completely absent from chromosome axes of *Syce3* ^−/−^ oocytes at both late pachytene- (median, 0) and dilpotene-like stage (median, 0) (Figure 9B). To rule out the possibility that the considerable alterations of RPA and MHL1 dynamics in SYCE3-deficient oocytes could be caused by a delay of meiotic progression per se, we quantified the ratio of pachytene (WT, n = 44; *Syce3^−/−^*, n = 53) and diplotene (WT, n = 33; *Syce3^−/−^*, n = 35) stages in 19.5 dpc oocytes of both *Syce3* ^−/−^ and wild-type females. Here, no significant difference could be observed (p = 0,688; Figure 9C).

Together, our data strongly argues that while progression through meiotic stages per se remains unaffected by the loss of SYCE3 in females, progression of recombination (i.e. processing of recombination intermediates into MLH1-marked late RNs, which are presumed markers of future crossovers in the wild-type) is critically depending on its presence.

### SYCE3 Is Part of a Central Element Protein Complex

**Figure 10:**
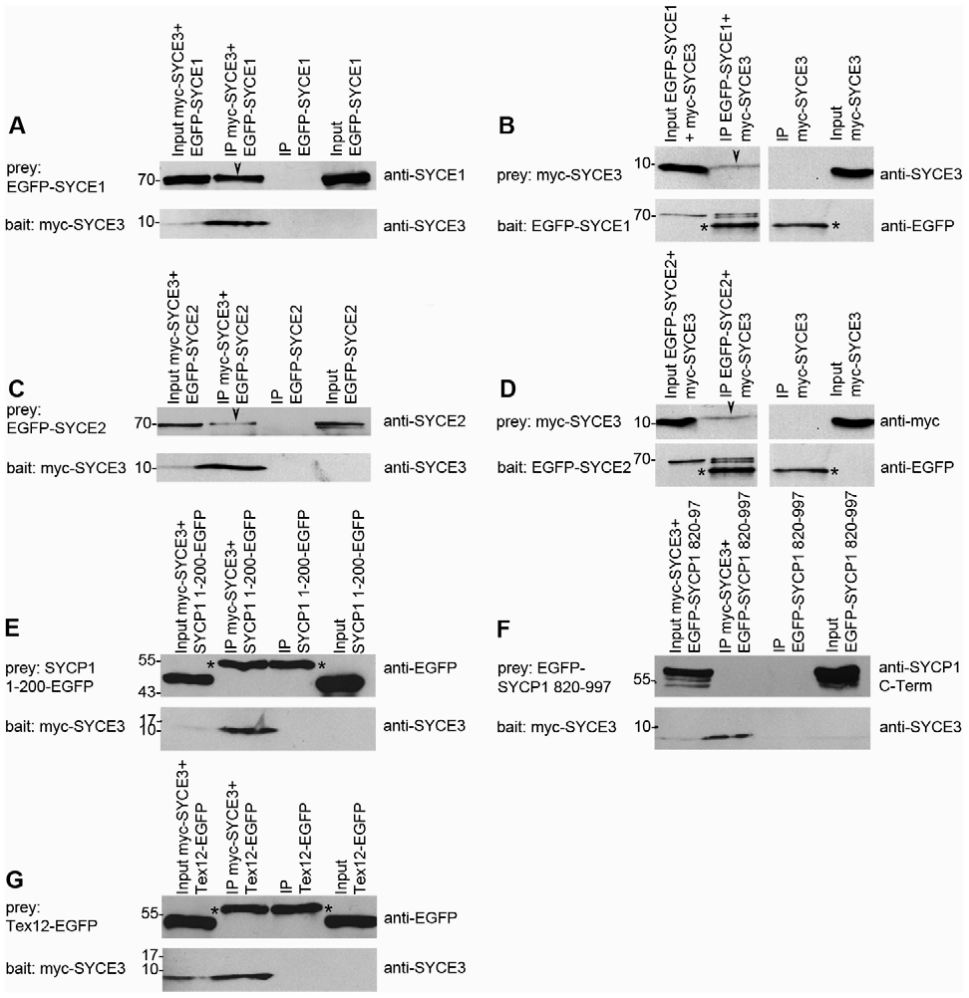
SYCE3 interacts with SYCE1 and SYCE2. Co-immunoprecipitation analysis was carried out by using transfected COS-7 cells. Cells were transfected with myc-SYCE3 and the respective EGFP fusion constructs of SYCE1, SYCE2, Tex12, SYCP1 1–200 and SYCP1 820–997. Protein-complexes were immunoprecipitated overnight with either an anti-myc antibody (A, C, E, F and G, bait: myc-SYCE3) or an anti-EGFP antibody (B, bait: EGFP-SYCE1; D, bait: EGFP-SYCE2) and analyzed by Western blot analysis. As a negative control, we performed an immunoprecipitation using the same antibody but cells that were solely transfected with the “prey” protein in parallel to each experiment. The input sample contained 5% of the total proteins used for the immunoprecipitation. Upper panels in each sub-figure show the detection of prey protein. Arrowheads indicate specific co-immunoprecipitated proteins. The lower part of each panel displays detection of the bait protein. Asterisks mark heavy chains of immunoglobulins (anti-myc or anti-EGFP antibody) utilized for immunoprecipitation. (A) myc-SYCE3 co-precipitates EGFP-SYCE1 highlighted by the arrowhead marking the SYCE1 specific band. (B) “Reverse” co-immunoprecipitation showing that EGFP-SYCE1 precipitates myc-SYCE3 (arrowhead marks myc-SYCE3). It can be seen in (C) that myc-SYCE3 pulls down EGFP-SYCE2 (arrowhead). (D) “Reverse” co-immunoprecipitation with EGFP-SYCE2 pulling down myc-SYCE3. (E–G) myc-SYCE3 does not bind to SYCP1 1-200-EGFP (E), EGFP-SYCP1 820–997 (F) or Tex12-EGFP (G).

We demonstrated that SYCE3 is important for fertility, initiation of synapsis and for a correct progression of meiotic recombination. Hence, we were interested in identifying binding partners of SYCE3 that would provide mechanistic insights into SYCE3 function. As the localization of the other CE components, SYCE1, SYCE2 and Tex12, is severely altered in *Syce3^−/−^* mice, they appear to be good candidates for being SYCE3 binding partners. Therefore, we performed co-transfection/immunoprecipitation experiments in somatic cells that do not express meiosis-specific proteins according to the approach described by Stewart-Hutchinson *et al.* [48]. COS-7 cells were transfected with EGFP- or myc-tagged fusion constructs of SYCE1, SYCE2, Tex12, SYCP1 N-terminus, SYCP1 C-terminus (as a control) and SYCE3. We used either a myc or an EGFP-specific antibody for precipitation. We found that SYCE3 interacts with SYCE1 (Figure 10A, 10B), which is consistent with the highly similar spatial-temporal expression of these two proteins. Interestingly, we show that SYCE3 also binds to SYCE2 although these two proteins do not exactly colocalize in pachytene spermatocytes (Figure 10C, 10D). However, under these experimental conditions, we could not detect an interaction between SYCE3 and Tex12 or the N- or C-terminus of SYCP1 (Figure 10E, 10F, 10G, see also below).

## Discussion

Meiotic chromosome synapsis is essential for proper meiotic progression as well as for male and female fertility. A critical step during this process is the assembly of the CE of the SC. In mammals, four proteins have been described which are essential for CE assembly: SYCP1, SYCE1, SYCE2 and Tex12. While SYCP1 is a component of the CE and TFs, the latter three proteins only localize to the CE [23], [24], [28], [29]. Here, we have identified SYCE3, a novel meiosis-specific protein that selectively localizes to the CE of the SC.

### SYCE3 Is Required for Initiation of Central Element Assembly and Chromosomal Loading of Central Element–Specific Proteins

Detailed analyses of *Syce2* and *Tex12* knockout mice showed that loss of each of these two genes causes infertility in both sexes. In both null mice, meiotic chromosomes align, and AEs form, but they do not synapse. Although synapsis between homologs is initiated at multiple positions along the axes, it fails to propagate along the entire chromosomes. SYCP1 and SYCE1 colocalize at these sites of synapsis initiation and, as revealed at the electron microscopical level, short CE-like structures become assembled. Furthermore, correct progression of meiotic recombination is altered in cells lacking SYCE2 or Tex12. Altogether, these data and the direct interaction of SYCE2 and Tex12 lead to the model that both SYCE2 and Tex12 are required for the longitudinal polymerization of SYCP1 filaments along the axial elements and thus for the propagation of synapsis along the homologs [31], [32].

Elimination of SYCE1 - the other currently known CE protein - displays a different phenotype. The absence of SYCE1 leads to the alignment of homologous chromosomes with a disrupted synapsis, but, in contrast to SYCE2 and Tex12 knockout mice, SYCE1-deficient mice display no sites of synapsis initiation and no CE-like structures at all. In these mice, SYCP1 is located in a weak discontinuous pattern along AEs, whether they are closely aligned or not. This indicates that under physiological conditions SYCP1 alone is insufficient for formation of stable head-to-head polymers for which CE proteins are required. Despite this striking difference to SYCE2 and Tex12 null mice, SYCE1-deficient mice also exhibit a disturbed progression of meiotic recombination [30].

Taken together, analysis of these central region knockout mice leads to three main conclusions: (1) The organization of the central region appears to be highly complex, (2) disruption of any currently known protein component causes defective synapsis leading to severe meiotic defects and infertility and (3) the correct assembly of the central region is also required for normal progression of meiotic recombination [27], [30]–[32].

Here, we have demonstrated that in wild-type cells SYCE3 distribution closely resembles SYCE1 localization (Figure 4; [28], [29]). Thus, we expected that SYCE3 is likely involved in a complex with SYCE1. Consistent with this assumption, no CE-like structures were detected in the EM analysis of *Syce3^−/−^* mouse testis sections (Figure 7B). The complete absence of any CE-like structure strongly suggests that, in contrast to SYCE2 and Tex12, SYCE3 is required for initiating synapsis. Furthermore, we have shown that SYCE1 and the SYCE2/Tex12 complex do not localize to chromosomes in *Syce3^−/−^* spermatocytes (Figure 7A). The lack of CE-like structures and CE-specific proteins indicates that SYCE3 is required upstream of SYCE1, SYCE2 and Tex12 and downstream of SYCP1 during the initiation of CE assembly. A possible function of SYCE3 could be to enable recruitment of SYCE1 and the SYCE2/Tex12 complex to SYCP1 N-termini. Loading of SYCE1 and SYCE2 to SYCP1 in turn would stabilize SYCP1 N-termini as proposed by Costa *et al.* [28]. This hypothesis is consistent with our results obtained from co-immunoprecipitation assays with transfected COS-7 cells, revealing that SYCE3 is capable of binding SYCE1 as well as SYCE2 (Figure 10). However, whether the role of SYCE3 in CE assembly is of a structural or regulatory character still needs to be clarified.

Taken together, we conclude that SYCE3 is essential for initiating synapsis and for chromosomal loading of SYCE1 and the SYCE2/Tex12 complex.

According to our present knowledge the behavior of SYCP1 and SYCE3 in meiotic cells lacking certain CE-specific proteins remains somewhat puzzling. In wild-type cells SYCP1 localization is restricted to synapsed chromosome axes. By contrast, in the absence of SYCE1, SYCP1 is observed in a weak, discontinuous pattern at the unsynapsed chromosome axes, no matter whether they were closely aligned or not. However, this is not the case in the absence of SYCE2 or Tex12, as under these conditions SYCP1 exclusively localizes to small foci at the sites of synapsis initiation [31]. These observations led to the hypothesis that SYCE1 is required to restrict SYCP1 to synapsed areas [30]. Our finding that SYCE3 also localizes to unsynapsed chromosome axes in SYCE1 null spermatocytes suggests that SYCE3 restriction to synapsed areas also depends on SYCE1 (Figure 5C). Nevertheless, we presently have no satisfactory explanation as to how this restricted localization can be accomplished. Another aspect that would require further investigation is the relationship between SYCP1 and SYCE3. As mentioned above, chromosomal loading of SYCE3 requires SYCP1. On the other hand, in our co-transfection/immunoprecipitation studies we obtained no evidence for an interaction between SYCP1 and SYCE3 (Figure 10). One possibility might be that interaction between these two proteins requires higher order structures of SYCP1 (for example dimerization or N-terminal association of dimers) that cannot occur under the conditions of the experimental assays. However, the existence of additional, yet undiscovered CE proteins that would mediate this binding cannot be ruled out a priori.

### Homologous Recombination Fails to Complete in the Absence of SYCE3

In early spermatocytes of *Syce3*-deficient mice γH2AX is distributed as in wild-type animals suggesting that homologous recombination is initiated in a wild-type manner. However, in later stages of prophase I γH2AX shows altered dynamics in spermatocytes and oocytes. In these cells it remains associated with chromosomes (Figure 8 and Figure 9). Thus we suggest that induced DSBs are repaired inefficiently in *Syce3* knockout meiocytes. This assumption is further supported by the analysis of components of the recombination machinery that assemble at the sites of DSBs as a further step during the process of meiotic recombination [41], [43]. In the absence of SYCE3, both *Syce3* ^−/−^ spermatocytes and oocytes reveal differences to the wild-type: RAD51 - as well as RPA foci - stay associated with chromosomal cores and virtually no MLH1 signal is detectable (Figure 8 and Figure 9).

The observed defects indicate that the exchange of homologous DNA strands (catalyzed by RAD51) and the formation of early recombination intermediates can take place in the absence of SYCE3, but further processing of the recombination sites fails to occur. As a consequence, no crossovers are formed in *Syce3* ^−/−^ meiocytes (see also [41], [43]). Interestingly, disruption of the CE by eliminating either *Syce1*, *Syce2* or *Tex12* results in a similar recombination phenotype. Our observations confirm and extend the notion that CE assembly and CE-specific protein components are not necessary for recombination initiation but essential for recombination progression [27], [30]–[32].

What is the physical link between the recombination machinery and the SC? In mammals, early recombination events and formation of ENs take place before SC central region assembly [27]. Early recombination events also appear to be independent of AE assembly during leptotene/zygotene as shown in mice lacking SYCP3. In these mice, AE assembly is impaired. However, they show normal levels of DMC1 foci formation and synapsis occurs between homologous chromosome regions [21], [22]. Recombination progression and crossover formation can also take place in the absence of AE assembly. As shown in female *Sycp3^−/−^* mice, oocytes reveal the presence of chiasmata but at a lower level than in the wild-type, which results in a reduction but not a complete loss of fertility [21]. In contrast, assembly of the SC central region is required for recombination progression and crossover formation as shown in mice lacking TF protein SYCP1 or any of the CE-specific proteins ([27], [30]–[32]; this study).

Previous electron microscopical studies revealed a close contact between RNs and SC components (e.g. [49], [50]). RNs seem to coalesce with the CE and fibers were shown to connect RNs and LEs [50]. These and other observations lead to the proposal that components of RNs might play a role in SC assembly. On the other hand, the SC central region can be seen as a platform for attachment and organization of RN components required for proper recombination progression (see [50], [30] and references therein). Our knowledge about the protein-protein interactions between components of the recombination machinery and the SC of mammals is rather fragmentary. Interactions have been reported between RAD51 and TF protein SYCP1 as well as CE protein SYCE2 [30], [51], and between TN component Tex11 (ZIP4H) and LE protein SYCP2 [52]. Although still preliminary, the emerging picture leads to the proposal that SCs and RNs are held together through a network of protein-protein interactions. Therefore, the recombination phenotype described here might be caused by the inability of RN components to bind SYCP1 and SYCE2 due to the defective localization and assembly of these proteins. However, a direct involvement in these interactions of additional CE element proteins (including SYCE3) cannot be excluded at present. Despite the progress in recent years, elucidation of the mutual dependence between SC assembly and meiotic recombination events would require additional experiments.

## Materials and Methods

### Ethics Statement

All animal care and experiments were conducted in accordance with the guidelines provided by the German Animal Welfare Act (German Ministry of Agriculture, Health and Economic Cooperation). For the generation of *Syce3* knockout mice we obtained approval from the Landesdirektion Dresden (24-9168.11-9/2005-1). Animal housing and breeding was approved by the regulatory agency of the city of Würzburg (Reference ABD/OA/Tr; according to §11/1 No. 1 of the German Animal Welfare Act). All aspects of the mouse work were carried out following strict guidelines to insure careful, consistent and ethical handling of mice.

### Primary Structure Analysis, Posttranslational Modifications, and Sequence Alignment

Taking the mouse SYCE3 protein sequence (GenBank accession number: NP_001156354) as query we analyzed predicted coiled-coil domains using PSORTII (http://psort.hgc.jp/) [53] and predicted phosphorylation sites (http://www.cbs.dtu.dk/services/NetPhos/) [54]. The mouse *Syce3* cDNA sequence also served as query for searching all GenBank sequences with the BlastN and TBlastN algorithm (http://blast.ncbi.nlm.nih.gov/Blast.cgi). Multiple sequence alignments were performed online with CLUSTALW (http://www.ebi.ac.uk/Tools/clustalw2/index.html) [55].

### RNA Extraction, Reverse Transcription, PCR, and cloning of SYCE3 cDNA

Whole RNA from mice testes from different ages and from various tissues of adult mice was extracted using peqGOLD TriFast™ (Peqlab, Erlangen, Germany) according to the manufacturer's protocol. cDNA was synthesized from 1 µg of RNA by reverse transcription with Oligo(dT) primers and M-MLV reverse transcriptase (Promega, Mannheim, Germany). Reverse transcribed cDNA samples were stored at −20°C before they were used in a polymerase chain reaction. Specific primers used for RT-PCRs and respective PCR conditions are listed in Table S1. In order to clone SYCE3 cDNA (GenBank accession number: HQ130280), we amplified full-length SYCE3 from cDNA derived from a reverse transcription of total testis RNA, as described above and using the same oligonucleotides and PCR conditions described for SYCE3 RT-PCR (Table S1).

### Antibody Generation

To raise SYCE3 specific antibodies, we generated and purified a GST-SYCE3 fusion protein using the vector pGEX-5X-1 (Amersham Pharmacia Biotech, Braunschweig, Germany) and the Bulk GST Purification Module (Amersham) according to the manufacturer's protocol. Anti-SYCE3 antisera were raised by immunizing a rabbit and a guinea pig with the purified GST-SYCE3 fusion protein (Seqlab, Göttingen, Germany). Specificity of both affinity-purified antibodies was validated by testing them on separate Western blots with protein lysates from wild-type, *Syce3^+/−^* and *Syce3^−/−^* littermates (generation of *Syce3^−/−^* mice is described below). The presence of the expected band of 12 kDa in wild-type and *Syce3^+/−^* testis lysates and moreover the absence of the aforementioned band in *Syce3^−/−^* testis lysates confirmed the specificity of both antibodies (see Figure S1A).

### SDS-PAGE, Tricine-SDS-PAGE, and Immunoblot Analysis

Protein samples derived from co-immunoprecipitation analysis were separated on 10%–15% polyacrylamide gels [56]. Separation of protein samples from testicular cells of adult mice was carried out by using tricine-SDS-PAGE (16% separating gel/6 M urea; [57]). Proteins were transferred to nitrocellulose membranes using the semi-dry Western blotting system described by Matsudaira [58]. The membranes were blocked overnight at 4°C in TBST buffer (10 mM Tris/HCl, pH 7.4, 150 mM NaCl, 0.1% Tween 20) containing 5% milk powder. Incubation with the respective primary antibody was carried out in blocking solution for 1 h at room temperature: guinea pig anti-SYCE3 (1∶1000), rabbit anti-SYCE3 (1∶1000), mouse anti-myc (1∶2000; R950-25, Invitrogen, Darmstadt, Germany), mouse anti-GFP (1∶200; sc-9996, Santa Cruz Biotechnology, Heidelberg, Germany), guinea pig anti-SYCE1 (1∶1000) [29], guinea pig anti-SYCE2 (1∶400) [29], mouse anti-actin (1∶10000, A4700, Sigma-Aldrich, Munich, Germany). Peroxidase-conjugated secondary antibodies were applied as specified by the manufacturer (Dianova, Hamburg, Germany). Bound antibodies were detected with the enhanced chemiluminescence system (Amersham). Western blots were stripped by incubating the membranes for 30 min in 0.1 M glycine buffer (pH 2.5) followed by a 30 min incubation in 0.1 M Tris buffer containing 2% SDS and subsequently washing three times in TBST.

### Immunocytochemistry and Histology

Spread-preparations of meiocytes were produced as described by de Boer *et al.* [59] and in each experiment spread-preparations were immunostained at the same time with the same mixture of the appropiate affinity-purified primary antibodies: rabbit anti-SYCE3 (1∶50), guinea pig anti-SYCE3 (1∶100), guinea pig anti-SYCE1 (1∶1000) [29], guinea pig anti-SYCE2 (1∶200) [29], guinea pig anti-SYCP1 (1∶150) [60], rabbit anti-SYCP1 (1∶200) [26], guinea pig anti-SYCP3 (1∶150) [61], rabbit anti-SYCP3 (1∶200; NB300-232, Acris, Herford, Germany) mouse anti-γH2AX (1∶500; 05-636, Millipore, Schwalbach/Ts., Germany), mouse anti-RPA (1∶40; NA19L, Calbiochem, Darmstadt, Germany), rabbit anti-RAD51 (1∶30; PC130, Calbiochem), mouse anti-MLH1 (1∶30; 551091, BD Pharmingen, Heidelberg, Germany). Secondary antibodies were applied as specified by the manufacturer (Dianova). Histology was performed on 5 µm sections of paraffin-embedded testis or ovary tissue fixed overnight in 4% formaldehyde according to standard protocols. Staging of mouse seminiferous tubule cross-sections was done according to Ahmed and de Rooij [62]. A TUNEL assay was carried out on 10 µm sections of paraffin embedded testis or ovary tissue fixed overnight in 4% formaldehyde using the ApopTag Peroxidase In Situ Apoptosis Kit (Millipore) according to the manufacturer's protocol.

### Statistical Analysis

Statistically significant differences in the medians of RPA and MLH1 foci comparing *Syce3^−/−^* and control cells were verified by Mann-Whitney *U* test. Numbers of pachytene and diplotene stages from control and *Syce3^−/−^* 19.5 dpc oocytes were statistically compared using a chi-square test.

### Microscopy and Imaging

Fluorescence microscopy was carried out by using a Zeiss Axiophot fluorescence microscope (Zeiss, Munich, Germany) equipped with a Plan-NEOFLUAR 40×/0.75 or a Plan-NEOFLUAR 20×/0.5 objective and the AxioCam MRm (Zeiss) camera. Digital images were pseudocoloured using the AxioVs40 V4.7.1.0 software release and processed using Adobe Photoshop (Adobe Systems, San Jose, CA). Light microscopy was carried out using the stereo microscope MZ FLIII (Leica). Confocal laser scanning microscopy was performed with a Leica TCS-SP2 confocal laser scanning microscope (Leica, Bensheim, Germany) equipped with a 63×/1.40 HCX PL APO lbd.BL oil-immersion objective. All confocal images are pseudocoloured using the Leica TCS-SP2 software and are two-dimensional projections calculated from a series of sequenced optical sections using the maximum projection algorithm (Leica). Imaging of wild-type and mutant SYCE1, SYCE2, SYCP1, SYCP3 and SYCE3 cells was performed using the same microscope settings. Digital images were processed with the same settings in Adobe Photoshop (Adobe Systems).

### Electron Microscopy

Electron microscopy was performed using ultra thin sections of testis tissue fixed in 2.5% glutaraldehyde and 1% osmium tetroxide as described previously [22]. For immunoelectron microscopy 10 µm cryosections of rat testis were fixed with acetone for 10 min at −20°C and air-dried. Incubation with primary antibodies (guinea pig anti-SYCE3 (1∶500–1∶1500); mouse anti-SYCP1 (1∶50) [26] was carried out in a humidified box for 4 h at room temperature. After rinsing twice in PBS, sections were fixed for 10 min in 2% formaldehyde and blocked with 50 mM NH_4_Cl. 6 nm gold conjugated secondary antibodies were incubated overnight at 4°C and samples were washed subsequently in PBS. Samples were fixed for 30 min in 2.5% glutaraldehyde and postfixed in 2% osmium tetroxide. After rinsing three times with H_2_O, samples were dehydrated in an ethanol series and embedded in Epon. Ultrathin sections were stained with uranyl acetate and lead citrate according to standard procedures [22].

### Generation of *Syce3* ^−/−^ Mice

We deleted the *Syce3* gene by replacing the entire coding sequence of SYCE3 (exons 2 and 3) with a neomycin cassette in reverse orientation using a modified pKS*loxP*NT vector. The vector for homologous recombination was designed as follows (see also Figure S2A): a 1.1 kbp genomic fragment (F1) containing part of intron 1 was cloned into the SalI restriction site downstream of the neomycin cassette and a 4.1 kbp fragment (F2) containing part of intron 3 was ligated into the EcoRI restriction site located in between the thymidine kinase and neomycin cassette. Electroporation of the modified replacement vector into the R1/E embryonic stem cells, laser assisted microinjection into 8-cell C57BL/6 morula and transfer of morula into CD1 (outbred) foster mice was performed at the transgenic core facility of the Max Planck Institute of Molecular Cell Biology and Genetics, Dresden. After electroporation and selection, we identified one positive ES cell clone by PCR with external primers (oligonucleotide sequence for genotyping: see Table S2) and confirmed correct targeting by Southern blot. For Southern blot analysis 10 µg BstEII digested DNA derived from ES cells (or tail tips of *Syce3^+/+^*, *Syce3^+/−^* and *Syce3^−/−^* mice) was loaded on a 0.8% agarose gel, subsequently transferred to a nylon membrane and correct insertion was tested with both external and neomycin probes. Blastocyst injection of the ES cell clone produced germline transmitting chimeras. Mating of chimeras with *C57BL*/6 mice gave rise to wild-type and *Syce3^+/−^* mice. Intercrossing of *Syce3^+/−^* mice produced offspring with all genotypes in Mendelian ratio. To confirm the absence of SYCE3 we performed PCR, Southern blot, immunofluorescence analysis on spread *Syce3^−/−^* spermatocytes and Western Blot analysis on *Syce3^−/∼^* testis tissue with polyclonal antibodies raised against the full-length SYCE3 protein (data not shown and Figure S1A).

### Transfection and Co-Immunoprecipitation

In order to express full-length SYCE3, SYCE1, SYCE2, Tex12 as well as SYCP1 N-terminal (aa 1–120) and SYCP1 C-terminal (aa 820–997) fusion constructs in the culture cell line COS-7 (green monkey kidney) for co-immunoprecipitation analysis, the respective cDNAs were inserted into pEGFP (Clontech, Heidelberg, Germany) or pCMV-Myc (BD Bioscience) vectors [60]. The fusion-constructs used (including the oligonucleotides used for cloning) are summarized in Table S3. Cells were transfected with the respective constructs using the effectene system according to the manufacturer's instructions (Qiagen, Hilden, Germany). Co-immunoprecipitation experiments were performed as described by Stewart-Hutchinson *et al.* [48] with the following modifications: (1) myc and EGFP constructs were immunoprecipitated with 0.5 µg of mouse anti-myc (R950-25, Invitrogen) or 0.5 µg of mouse anti-GFP (sc-9996, Santa Cruz Biotechnology) antibody per 60-mm dish. (2) Immune complexes were pulled down by protein G dynabeads (100-03D, Invitrogen).

## Supporting Information

Figure S1 SYCE3 is expressed in mouse testis. (A) Specificity of affinity purified anti-SYCE3 antibodies. Western blot analysis of *Syce3^+/+^*, *Syce3^+/−^* and *Syce3^−/−^* testis tissue separated on a 16%/6 M urea tricin-SDS gel and detection of SYCE3 with an affinity purified rabbit anti-SYCE3 (left) and an affinity purified guinea pig anti-SYCE3 antibody (right). The same Western blots were stripped and incubated with a mouse anti-actin antibody as a loading control. (B) SYCE3 localization is restricted to meiotic cells. Immunolocalization of SYCE3 (green) on frozen sections of an adult wild-type mouse testis. DNA is labeled with Hoechst. (eSp) elongated spermatids, (Sc) spermatocytes, (Sg) spermatogonia, (Ser) Sertoli cells. Bar, 50 µm.
(TIF)

Figure S2 Generation and characterization of a *Syce3^−/−^* mouse. (A) Structures of the *Syce3* gene located on chromosome 15, the replacement vector and the mutant containing the neomycin cassette. The location of the Southern blot probe and the lengths of expected fragments after BstEII digestion of wild-type and mutant samples is depicted below. (B) Long arm (F2) and short arm (F1) PCR of positively tested ES cell clone using external oligonucleotides. (C) Southern blot of a wild-type and positively tested ES cell clone with a SYCE3 specific external probe (left) and neomycin-specific probe (right). (D) Correct insertion of the replacement vector and genotyping of *Syce3^−/−^* mice was confirmed by Southern blot analysis using a SYCE3 specific external probe (left) and neomycin-specific probe (right). (E) Testes from *Syce3^+/+^* (left) and *Syce3^−/−^* (right) littermates. Bar, 200 µm.
(TIF)

Figure S3 Loss of SYCE3 results in massive apoptotic events during spermatogenesis. (A) TUNEL assay on paraffin embedded testis sections from wild-type, heterozygote and homozygote *Syce3^−/−^* mice (day 30). TUNEL positive stained cells are labeled in green, DNA is shown in blue. Bar, 40 µm. (B) Light microscopic images of testis sections from paraffin embedded *Syce3^−/−^* mice showing stage X-II, IV and V–VI tubules. In stage X-II type A spermatogonia and zygotene or pachytene spermatocytes are present. Stage IV is characterized by intermediate spermatogonia and apoptotic pachytene spermatocytes. In type V–VI, B type spermatogonia can be distinguished. (Ser) Sertoli cells, (P) pachytene cells, (A) type A spermatogonia, (aP) apoptotic pachytene cells, (In) intermediate spermatogonia and (B) type B spermatogonia. Bar, 10 µm.
(TIF)

Figure S4 SYCP1 localization in *Syce1^−/−^* and *Syce2^−/−^* spermatocytes. Immunofluorescence analysis of spread preparations of wild-type and (A) *Syce1^−/−^* or (B) *Syce2^−/−^* mouse spermatocytes stained with SYCP1 and SYCP3. As previously described, SYCP1 localizes to *Syce1^−/−^* AEs in a weak discontinuous pattern (A and [30]), whereas SYCP1-staining is confined to sites of closer association of homologs in *Syce2^−/−^* spermatocytes (B, and [31]). These results clearly demonstrate that under our experimental conditions we can reproduce previously described weak immunofluorescence signals, consequently allowing a precise comparison of different CE-mutant phenotypes. Bar, 10 µm.
(TIF)

Table S1 Sequence of primers and PCR conditions used for RT-PCR.
(DOC)

Table S2 Sequence of primers used for genotyping electroporated R1/E cells and SYCE3 knockout mice.
(DOC)

Table S3 EGFP- and myc-fusion constructs used for co-immunoprecipitation analysis. Columns 2 and 3 show primers used for cloning and column 4 the target vector.
(DOC)

## References

[1] Zickler D, Kleckner N (1999). Meiotic chromosomes: integrating structure and function.. Annu Rev Genet.

[2] Page SL, Hawley RS (2004). The genetics and molecular biology of the synaptonemal complex.. Annu Rev Cell Dev Biol.

[3] Yang F, Wang PJ (2009). The mammalian synaptonemal complex: a scaffold and beyond.. Genome Dyn.

[4] Roeder GS (1995). Sex and the single cell: meiosis in yeast.. Proc Natl Acad Sci U S A.

[5] Colaiácovo MP (2006). The many facets of SC function during C. elegans meiosis.. Chromosoma.

[6] Kleckner N (2006). Chiasma formation: chromatin/axis interplay and the role(s) of the synaptonemal complex.. Chromosoma.

[7] Zickler D (2006). From early homologue recognition to synaptonemal complex formation.. Chromosoma.

[8] Bhalla N, Dernburg AF (2008). Prelude to a division.. Annu Rev Cell Dev Biol.

[9] McKee BD (2009). Homolog pairing and segregation in Drosophila meiosis.. Genome Dyn.

[10] Zetka M (2009). Homologue pairing, recombination and segregation in Caenorhabditis elegans.. Genome Dyn.

[11] Costa Y, Cooke H (2007). Dissecting the mammalian synaptonemal complex using targeted mutations.. Chromosome Res.

[12] Handel MA, Schimenti JC (2010). Genetics of mammalian meiosis: regulation, dynamics and impact on fertility.. Nat Rev Genet.

[13] Miyamoto T, Hasuike S, Yogev L, Maduro MR, Ishikawa M (2003). Azoospermia in patients heterozygous for a mutation in SYCP3.. Lancet.

[14] Baier A, Alsheimer M, Benavente R (2007). Synaptonemal complex protein SYCP3: conserved polymerization properties among vertebrates.. Biochim Biophys Acta.

[15] Bolor H, Mori T, Nishiyama S, Ito Y, Hosoba E (2009). Mutations of the *SYCP3* gene in women with recurrent pregnancy loss.. Am J Hum Genet.

[16] Zickler D, Kleckner N (1998). The leptotene–zygotene transition of meiosis.. Annu Rev Genet.

[17] Pelttari J, Hoja MR, Yuan L, Liu JG, Brundell E (2001). A meiotic chromosomal core consisting of cohesin complex proteins recruits DNA recombination proteins and promotes synapsis in the absence of an axial element in mammalian meiotic cells.. Mol Cell Biol.

[18] Prieto I, Suja JA, Pezzi N, Kremer L, Martínez-A C (2001). Mammalian STAG3 is a cohesin specific to sister chromatid arms in meiosis I.. Nat Cell Biol.

[19] Eijpe M, Offenberg H, Jessberger R, Revenkova E, Heyting C (2003). Meiotic cohesin REC8 marks the axial elements of rat synaptonemal complexes before cohesins SMC1β and SMC3.. J Cell Biol.

[20] Yuan L, Liu J, Zhao J, Brundell E, Daneholt B (2000). The murine *SCP3* gene is required for synaptonemal complex assembly, chromosome synapsis, and male fertility.. Mol Cell.

[21] Yuan L, Liu J, Hoja M, Wilbertz J, Nordqvist K (2002). Female germ cell aneuploidy and embryo death in mice lacking the meiosis-specific protein SCP3.. Science.

[22] Liebe B, Alsheimer M, Höög C, Benavente R, Scherthan H (2004). Telomere attachment, meiotic chromosome condensation, pairing, and bouquet stage duration are modified in spermatocytes lacking axial elements.. Mol Biol Cell.

[23] Meuwissen RLJ, Offenberg HH, Dietrich AJJ, Riesewijk A, van Iersel M (1992). A coiled-coil related protein specific for synapsed regions of meiotic prophase chromosomes.. EMBO J.

[24] Liu JG, Yuan L, Brundell E, Björkroth B, Daneholt B (1996). Localization of the N-terminus of SCP1 to the central element of the synaptonemal complex and evidence for direct interactions between the N-termini of SCP1 molecules organized head-to-head.. Exp Cell Res.

[25] Schmekel K, Meuwissen RL, Dietrich AJJ, Vink ACG, van Marle J (1996). Organization of SCP1 protein molecules within synaptonemal complexes of the rat.. Exp Cell Res.

[26] Ollinger R, Alsheimer M, Benavente R (2005). Mammalian protein SCP1 forms synaptonemal complex-like structures in the absence of meiotic chromosomes.. Mol Biol Cell.

[27] de Vries FA, de Boer E, van den Bosch M, Baarends WM, Ooms M (2005). Mouse *Sycp1* functions in synaptonemal complex assembly, meiotic recombination, and XY body formation.. Genes Dev.

[28] Costa Y, Speed R, Ollinger R, Alsheimer M, Semple CA (2005). Two novel proteins recruited by synaptonemal complex protein 1 (SYCP1) are at the centre of meiosis.. J Cell Sci.

[29] Hamer G, Gell K, Kouznetsova A, Novak I, Benavente R (2006). Characterization of a novel meiosis-specific protein within the central element of the synaptonemal complex.. J Cell Sci.

[30] Bolcun-Filas E, Hall E, Speed R, Taggart M, Grey C (2009). Mutation of the mouse *Syce1* gene disrupts synapsis and suggests a link between synaptonemal complex structural components and DNA repair.. PLoS Genet.

[31] Bolcun-Filas E, Costa Y, Speed R, Taggart M, Benavente R (2007). SYCE2 is required for synaptonemal complex assembly, double strand break repair, and homologous recombination.. J Cell Biol.

[32] Hamer G, Wang H, Bolcun-Filas E, Cooke HJ, Benavente R (2008). Progression of meiotic recombination requires structural maturation of the central element of the synaptonemal complex.. J Cell Sci.

[33] Romanienko PJ, Camerini-Otero RD (2000). The mouse *Spo11* gene is required for meiotic chromosome synapsis.. Mol Cell.

[34] Baudat F, Manova K, Yuen JP, Jasin M, Keeney S (2000). Chromosome synapsis defects and sexually dimorphic meiotic progression in mice lacking SPO11.. Mol Cell.

[35] Maratou K, Forster T, Costa Y, Taggart M, Speed R (2004). Expression profiling of the developing testis in wild-type and *Dazl* knockout mice.. Mol Reprod Dev.

[36] Moens PB, Heyting C, Dietrich AJJ, van Raamsdonk W, Chen Q (1987). Synaptonemal complex antigen location and conservation.. J Cell Biol.

[37] Kranz A, Fu J, Duerschke K, Weidlich S, Naumann R (2010). An improved Flp deleter mouse in C57Bl/6 based on Flpo recombinase.. Genesis.

[38] Mahadevaiah SK, Turner JM, Baudat F, Rogakou EP, de Boer P (2001). Recombinational DNA double-strand breaks in mice precede synapsis.. Nat Genet.

[39] Albini SM, Jones GH (1987). Synaptonemal complex spreading in *Allium cepa* and *A. fistulosum*.. Chromosoma.

[40] Barlow AL, Benson FE, West SC, Hultén MA (1997). Distribution of the Rad51 recombinase in human and mouse spermatocytes.. EMBO J.

[41] Moens PB, Kolas NK, Tarsounas M, Marcon E, Cohen PE (2002). The time course and chromosomal localization of recombination-related proteins at meiosis in the mouse are compatible with models that can resolve the early DNA-DNA interactions without reciprocal recombination.. J Cell Sci.

[42] Carpenter AT (1975). Electron microscopy of meiosis in Drosophila melanogaster females: II. The recombination nodule–a recombination-associated structure at pachytene?. Proc Natl Acad Sci USA.

[43] Moens PB, Marcon E, Shore JS, Kochakpour N, Spyropoulos B (2007). Initiation and resolution of interhomolog connections: crossover and non-crossover sites along mouse synaptonemal complexes.. J Cell Sci.

[44] Marcon E, Moens P (2003). MLH1p and MLH3p localize to precociously induced chiasmata of okadaic-acid-treated mouse spermatocytes.. Genetics.

[45] Baker SM, Plug AW, Prolla TA, Bronner CE, Harris AC (1996). Involvement of mouse *Mlh1* in DNA mismatch repair and meiotic crossing over.. Nat Genet.

[46] Borum K (1961). Oogenesis in the mouse. A study of the meiotic prophase.. Exp Cell Res.

[47] Speed RM (1982). Meiosis in the foetal mouse ovary. I. An analysis at the light microscope level using surface-spreading.. Chromosoma.

[48] Stewart-Hutchinson PJ, Hale CM, Wirtz D, Hodzic D (2008). Structural requirements for the assembly of LINC complexes and their function in cellular mechanical stiffness.. Exp Cell Res.

[49] Rasmussen SW, Holm PB (1978). Human meiosis II. Chromosome pairing and recombination nodules in human spermatocytes.. Carlsberg Res Commun.

[50] Schmekel K, Daneholt B (1998). Evidence for close contact between recombination nodules and the central element of the synaptonemal complex.. Chromosome Res.

[51] Tarsounas M, Morita T, Pearlman RE, Moens PB (1999). RAD51 and DMC1 form mixed complexes associated with mouse meiotic chromosome cores and synaptonemal complexes.. J Cell Biol.

[52] Yang F, Gell K, van der Heijden GW, Eckardt S, Leu NA (2008). Meiotic failure in male mice lacking an X-linked factor.. Genes Dev.

[53] Schultz J, Copley RR, Doerks T, Ponting CP, Bork P (2000). SMART: A Web-based tool for the study of genetically mobile domains.. Nucleic Acids Res.

[54] Blom N, Gammeltoft S, Brunak S (1999). Sequence and structure-based prediction of eukaryotic protein phosphorylation sites.. J Mol Biol.

[55] Larkin MA, Blackshields G, Brown NP, Chenna R, McGettigan PA (2007). Clustal W and Clustal X version 2.0.. Bioinformatics.

[56] Laemmli UK (1970). Cleavage of structural proteins during the assembly of the head of bacteriophage T4.. Nature.

[57] Schagger H (2006). Tricine-SDS-PAGE.. Nat Protoc.

[58] Matsudaira P (1987). Sequence from picomole quantities of proteins electroblotted onto polyvinylidene difluoride membranes.. J Biol Chem.

[59] de Boer E, Lhuissier FGP, Heyting C (2009). Cytological analysis of interference in mouse meiosis.. Meiosis.

[60] Winkel K, Alsheimer M, Öllinger R, Benavente R (2009). Protein SYCP2 provides a link between transverse filaments and lateral elements of mammalian synaptonemal complexes.. Chromosoma.

[61] Alsheimer M, Benavente R (1996). Change of karyoskeleton during mammalian spermatogenesis: expression pattern of nuclear lamin C2 and its regulation.. Exp Cell Res.

[62] Ahmed AE, de Rooij DG (2009). Staging of mouse seminiferous tubule cross-sections.. Meiosis.

