# Supplementary figures and images for: A Novel Mouse Synaptonemal Complex Protein Is Essential for Loading of Central Element Proteins, Recombination, and Fertility
Source: PLoS Genet. 2011 May 26;7(5):e1002088. doi: 10.1371/journal.pgen.1002088 (PMC3102746; doi:10.1371/journal.pgen.1002088)

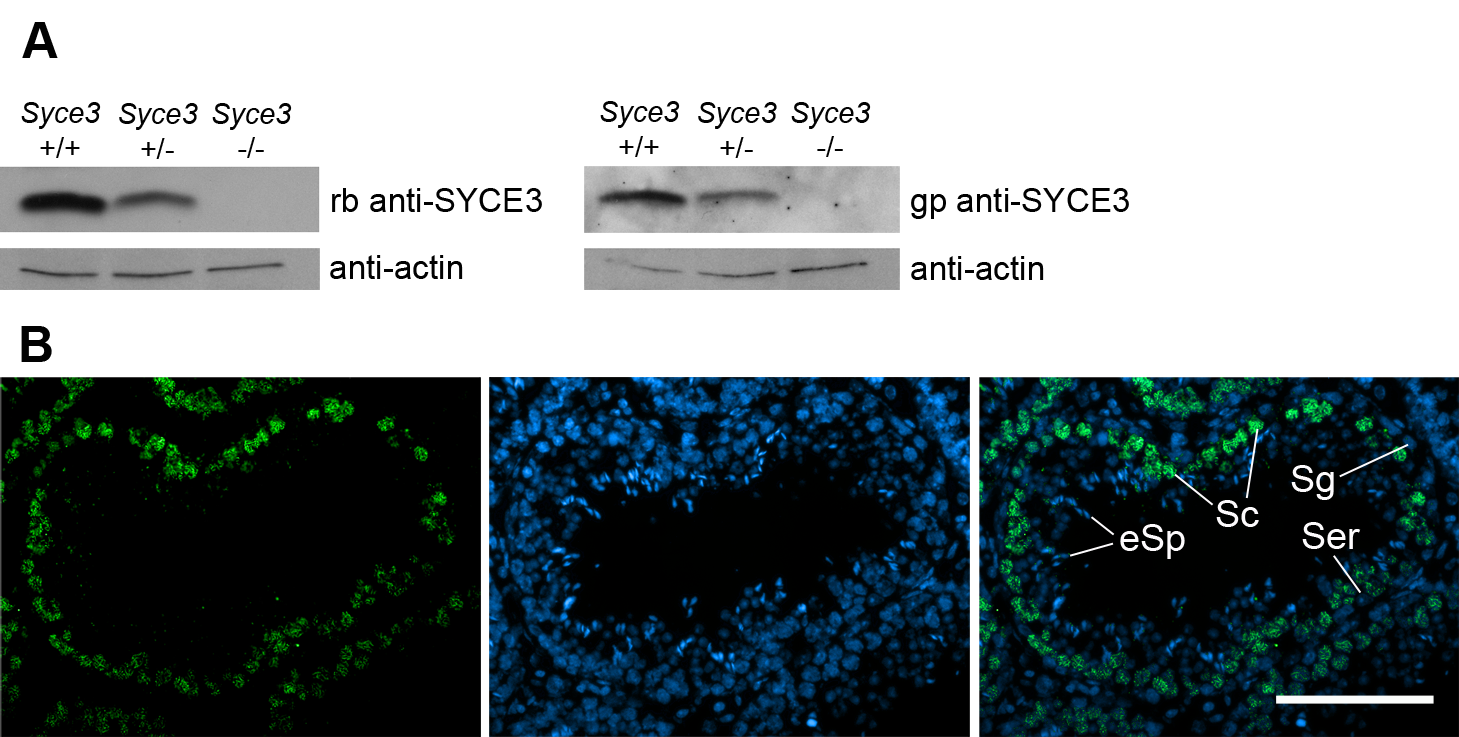

Supplement: Figure S1 — SYCE3 is expressed in mouse testis. (A) Specificity of affinity purified anti-SYCE3 antibodies. Western blot analysis of Syce3+/+, Syce3+/− and Syce3−/− testis tissue separated on a 16%/6 M urea tricin-SDS gel and detection of SYCE3 with an affinity purified rabbit anti-SYCE3 (left) and an affinity purified guinea pig anti-SYCE3 antibody (right). The same Western blots were stripped and incubated with a mouse anti-actin antibody as a loading control. (B) SYCE3 localization is restricted to meiotic cells. Immunolocalization of SYCE3 (green) on frozen sections of an adult wild-type mouse testis. DNA is labeled with Hoechst. (eSp) elongated spermatids, (Sc) spermatocytes, (Sg) spermatogonia, (Ser) Sertoli cells. Bar, 50 µm. (TIF) [file pgen.1002088.s001.tif]

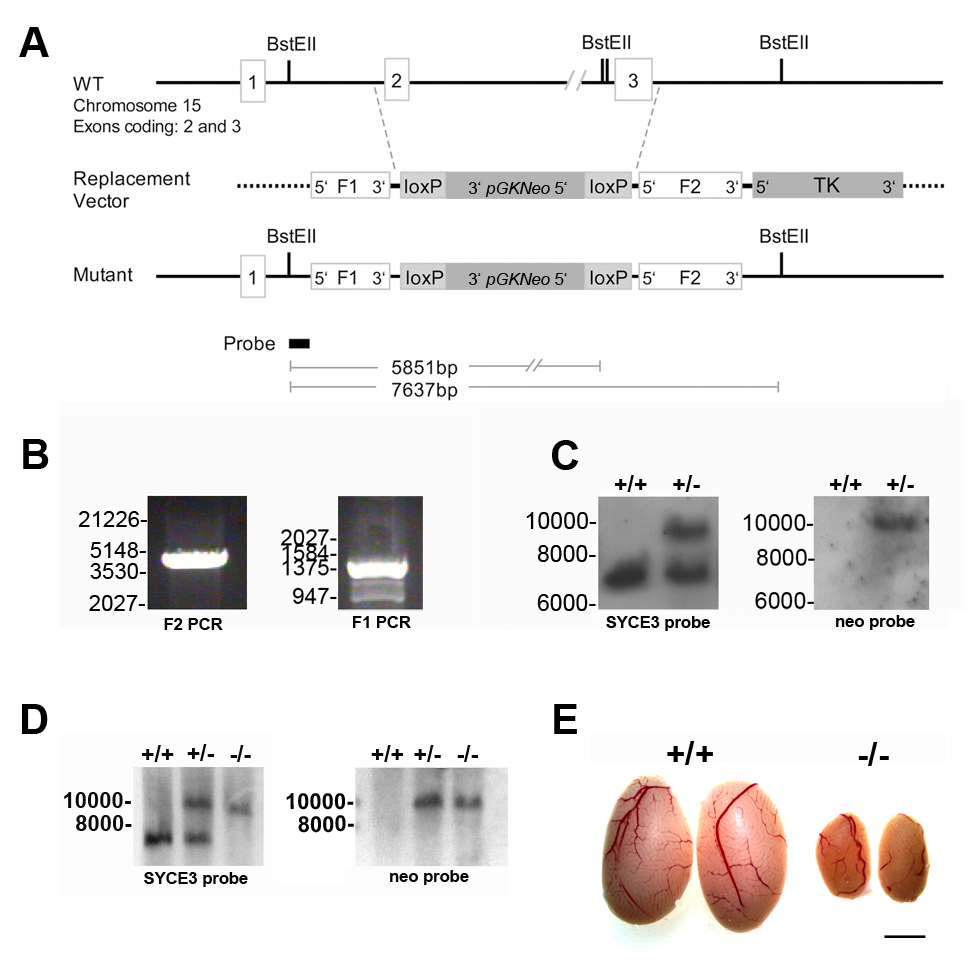

Supplement: Figure S2 — Generation and characterization of a Syce3−/− mouse. (A) Structures of the Syce3 gene located on chromosome 15, the replacement vector and the mutant containing the neomycin cassette. The location of the Southern blot probe and the lengths of expected fragments after BstEII digestion of wild-type and mutant samples is depicted below. (B) Long arm (F2) and short arm (F1) PCR of positively tested ES cell clone using external oligonucleotides. (C) Southern blot of a wild-type and positively tested ES cell clone with a SYCE3 specific external probe (left) and neomycin-specific probe (right). (D) Correct insertion of the replacement vector and genotyping of Syce3−/− mice was confirmed by Southern blot analysis using a SYCE3 specific external probe (left) and neomycin-specific probe (right). (E) Testes from Syce3+/+ (left) and Syce3−/− (right) littermates. Bar, 200 µm. (TIF) [file pgen.1002088.s002.tif]

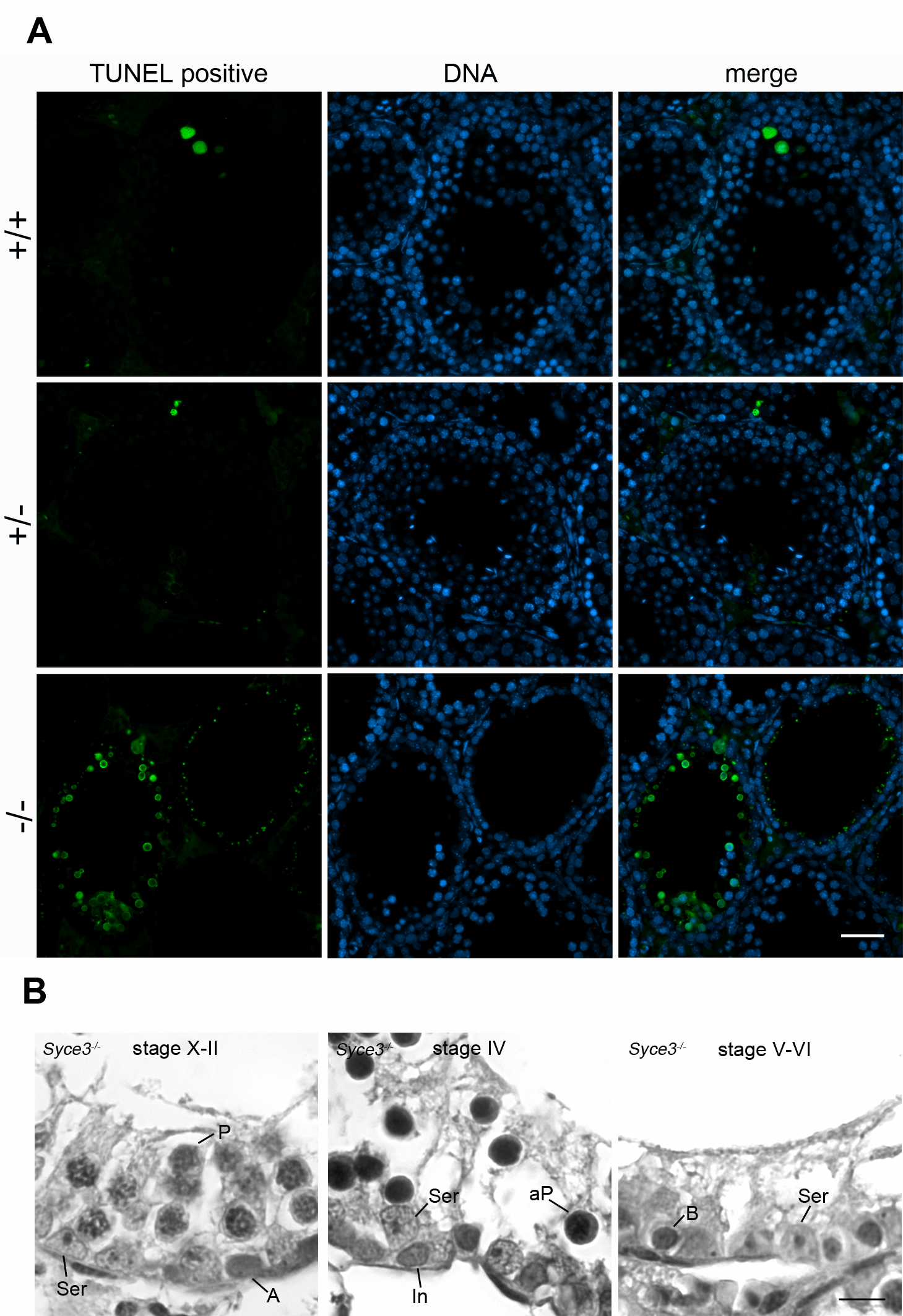

Supplement: Figure S3 — Loss of SYCE3 results in massive apoptotic events during spermatogenesis. (A) TUNEL assay on paraffin embedded testis sections from wild-type, heterozygote and homozygote Syce3−/− mice (day 30). TUNEL positive stained cells are labeled in green, DNA is shown in blue. Bar, 40 µm. (B) Light microscopic images of testis sections from paraffin embedded Syce3−/− mice showing stage X-II, IV and V–VI tubules. In stage X-II type A spermatogonia and zygotene or pachytene spermatocytes are present. Stage IV is characterized by intermediate spermatogonia and apoptotic pachytene spermatocytes. In type V–VI, B type spermatogonia can be distinguished. (Ser) Sertoli cells, (P) pachytene cells, (A) type A spermatogonia, (aP) apoptotic pachytene cells, (In) intermediate spermatogonia and (B) type B spermatogonia. Bar, 10 µm. (TIF) [file pgen.1002088.s003.tif]

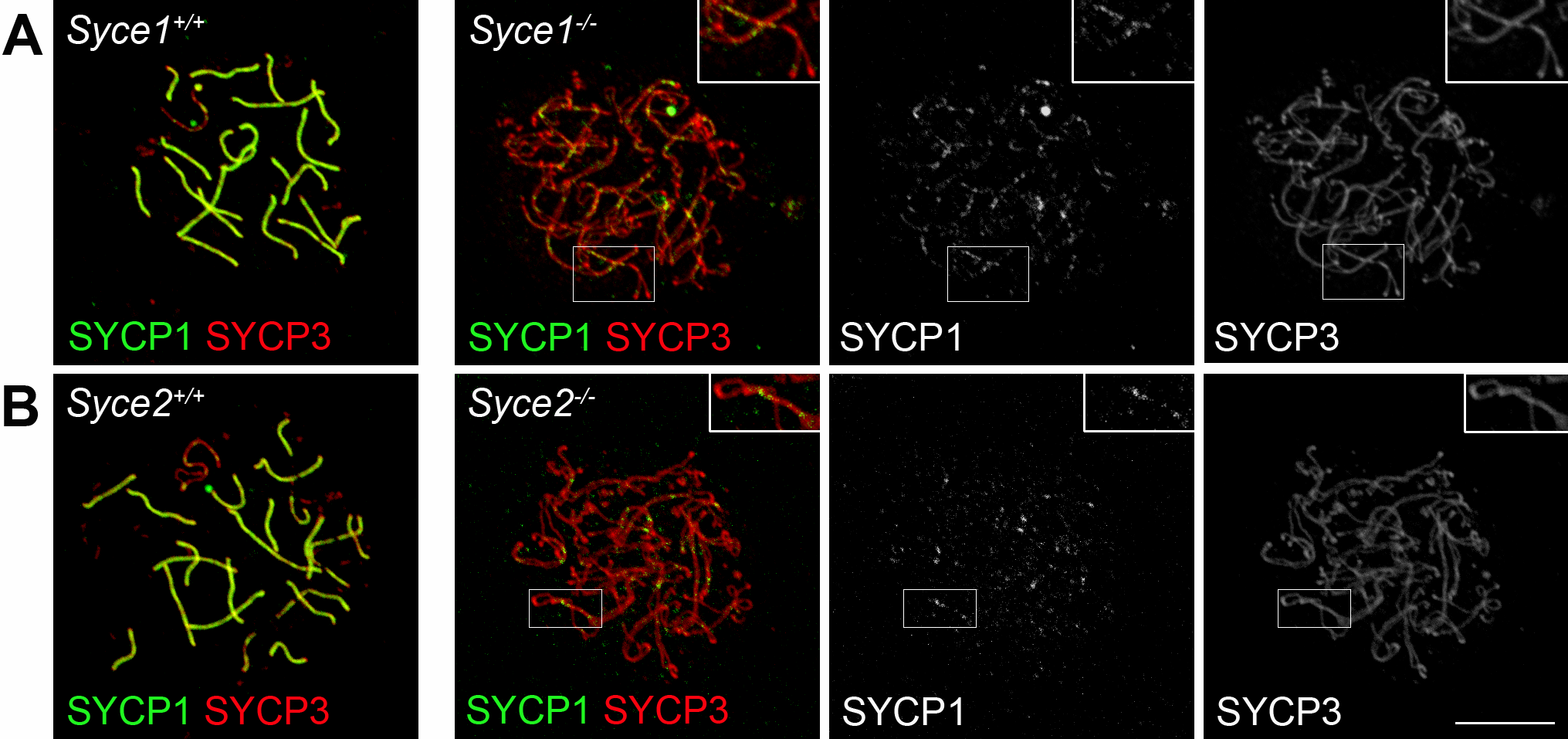

Supplement: Figure S4 — SYCP1 localization in Syce1−/− and Syce2−/− spermatocytes. Immunofluorescence analysis of spread preparations of wild-type and (A) Syce1−/− or (B) Syce2−/− mouse spermatocytes stained with SYCP1 and SYCP3. As previously described, SYCP1 localizes to Syce1−/− AEs in a weak discontinuous pattern (A and [30]), whereas SYCP1-staining is confined to sites of closer association of homologs in Syce2−/− spermatocytes (B, and [31]). These results clearly demonstrate that under our experimental conditions we can reproduce previously described weak immunofluorescence signals, consequently allowing a precise comparison of different CE-mutant phenotypes. Bar, 10 µm. (TIF) [file pgen.1002088.s004.tif]
