# Supplementary material for: A Novel Mouse Synaptonemal Complex Protein Is Essential for Loading of Central Element Proteins, Recombination, and Fertility
Source: PLoS Genet. 2011 May 26;7(5):e1002088. doi: 10.1371/journal.pgen.1002088 (PMC3102746; doi:10.1371/journal.pgen.1002088)
Supplement: Table S1 — Sequence of primers and PCR conditions used for RT-PCR. (DOC) [file pgen.1002088.s005.doc]

Table S1:

|  | **Forward Primer 5’-3’** | **Reverse Primer 3’-5’** | **Annealing** | **Cycles** |
| --- | --- | --- | --- | --- |
| **SYCE3** | ATGGCTGATTCCGATCCTGGG | GTACCATGCACATGGCTACACGTCT | 65°C | 25 |
| **SYCP3** | GGCTTCGTCAGATGCTTCGAG | GACTCATCAGAATAACATGGATTGAAG | 52°C | 25 |
| **GAPDH** | GGGCCCACTTGAAGGGTGGAGC | GTCAGATCCACGACGGACACATTGG | 58°C | 25 |
