# Supplementary material for: A Novel Mouse Synaptonemal Complex Protein Is Essential for Loading of Central Element Proteins, Recombination, and Fertility
Source: PLoS Genet. 2011 May 26;7(5):e1002088. doi: 10.1371/journal.pgen.1002088 (PMC3102746; doi:10.1371/journal.pgen.1002088)
Supplement: Table S2 — Sequence of primers used for genotyping electroporated R1/E cells and SYCE3 knockout mice. (DOC) [file pgen.1002088.s006.doc]

Table S2:

|  | **Forward Primer 5’-3’** | **Reverse Primer 3’-5’** | **Annealing** | **Cycles** |
| --- | --- | --- | --- | --- |
| **SYCE3 F1 (1502bp)** | CAGGTGAAAGGTGAGGGCCTG | CGAAGCTTATCGATACCGTCGAC | 65°C | 35 |
| **SYCE3 F2 (4271bp)** | GCTGGACGTAAACTCCTCTTCAGAC | GGAGAGGTGACCACCAGAGGG | 63°C | 35 |
