# Supplementary material for: A Novel Mouse Synaptonemal Complex Protein Is Essential for Loading of Central Element Proteins, Recombination, and Fertility
Source: PLoS Genet. 2011 May 26;7(5):e1002088. doi: 10.1371/journal.pgen.1002088 (PMC3102746; doi:10.1371/journal.pgen.1002088)
Supplement: Table S3 — EGFP- and myc-fusion constructs used for co-immunoprecipitation analysis. Columns 2 and 3 show primers used for cloning and column 4 the target vector. (DOC) [file pgen.1002088.s007.doc]

Table S3:

| **Construct name** | **Forward Primer 5’-3’** | **Reverse Primer 3’-5’** | **Vector** |
| --- | --- | --- | --- |
| **EGFP-SYCE3** | ATGGCTGATTCCGATCCTGGG | GTACCATGCACATGGCTACACGTCT | EGFP C2 |
| **EGFP-SYCE1** | ATGGCCACCAGACCGCA | TTAGGTCCTGCTTGATGGGCG | EGFP C2 |
| **EGFP-SYCE2** | ATGGAGCGCCACGGAGTG | TCAGCATTCACCATCTTTGTAATTCT | EGFP C2 |
| **Tex12-EGFP** | ATGATGGCAAACCACCTTGTAA | CTTGTGTAGGGTGTTGGTAATTACTGT | EGFP N2 |
| **SYCP1 1-200-EGFP** | GAAGCGCTCGAGGGCACGGAG | CCACTTTTTTATCTTTTCAGCCTCTTTATACAGC | EGFP N2 |
| **EGFP-SYCP1 820 - 997** | TCACCTGAAGCCACTAGTTGGAAAT | TTAAGTAAATAACTTTTCTGCTTCCTTTAGTCTTC | EGFP C2 |
| **Myc-SYCE3** | TCGAATTCGGATGGCTGATTCCGATC | TTGTCGACGTACCATGCACATGGCTACAC | pCMV myc HA |
